# Prolonged Visual Entrainment Induces Long‐Lasting Alpha‐Band Modulations

**DOI:** 10.1111/psyp.70321

**Published:** 2026-05-17

**Authors:** Gianluca Marsicano, Jessica Gallina, Sara Giovagnoli, Luca Ronconi, Caterina Bertini

**Affiliations:** ^1^ Department of Psychology University of Bologna Bologna Italy; ^2^ Centre for Studies and Research in Cognitive Neuroscience University of Bologna Cesena Italy; ^3^ Department of Psychology and Cognitive Science University of Trento Rovereto Italy

**Keywords:** alpha oscillations, EEG, entrainment, neural oscillations, sensory stimulation, theta oscillations

## Abstract

Neural oscillations are fundamental for encoding, filtering, and integrating sensory information, representing a core computational principle underlying perceptual experience. In particular, fluctuations in alpha (~7–13 Hz) and theta (~4–7 Hz) activity are associated with changes in cortical excitability and rhythmic modulations of perception. These oscillations are not static features but are highly plastic and can be shaped through neural entrainment, whereby brain rhythms synchronize with external rhythmic sensory stimulation. While short‐duration (~0.5–5 s) stimulation induces transient (~500 ms), localized entrainment effects, it remains unclear whether prolonged entrainment can produce persistent and spatially widespread modulations in ongoing neural activity. Here, we recorded resting‐state EEG before and after 1 min of visual rhythmic stimulation at individualized alpha (individual alpha frequency, IAF; IAF −2 Hz; IAF +2 Hz) and theta (4.5 Hz) frequencies. We assessed frequency‐specific and topographic effects by comparing alpha (7–13 Hz) and theta (3–6 Hz) power, frequency, and phase coherence before and after stimulation. Our results show that prolonged visual entrainment induces sustained increases in alpha power and phase coherence, persisting throughout the 1‐min post‐stimulation period without changes in frequency. Stimulation at IAF induced stronger modulations of intrinsic alpha oscillations than other frequencies, extending bilaterally from posterior to central and anterior regions. In contrast, theta stimulation increased phase consistency but did not induce persistent changes in theta power, suggesting no sustained modulation of endogenous theta activity. Together, these findings provide evidence that prolonged visual entrainment can induce long‐lasting and spatially distributed modulations of resting‐state alpha oscillations, particularly when stimulation is tuned to the brain's intrinsic resonant frequency. This highlights the frequency‐specific nature of neural responsiveness and the potential of rhythmic sensory stimulation to induce long‐lasting changes in large‐scale brain oscillatory networks.

## Impact Statement

This study demonstrates that prolonged visual entrainment at alpha frequencies can induce sustained, frequency‐specific changes in resting‐state alpha brain activity. By showing that entrainment effects are strongest when stimulation matches an individual's intrinsic frequency, our findings provide new evidence for resonance‐driven plasticity in large‐scale neural networks. These results advance current understanding of how sensory rhythms can durably shape brain oscillatory dynamics, with implications for non‐invasive modulation of cognitive and perceptual functions.

## 1 Introduction

Rhythmicity is an intrinsic property of neural activity, crucial for the functional coordination with the perceptual environment and for organizing the temporal structure of human cognition (Haegens and Zion Golumbic 2018).

Among all spontaneous neural rhythms, converging evidence suggests that the neural rhythmicity observable in the alpha‐band (~7–13 Hz), which is prominently distributed over occipito‐parietal cortices, may be responsible for balancing sensory cortices excitability and organizing perceptual performance (Romei et al. 2008; VanRullen 2016; Cuello et al. 2022; Fakche and Dugué 2024; Wutz 2024; Gallina et al. 2024). Historically, alpha brain activity has been considered as an “idling” rhythm, predominantly observed in the resting awake brain, and thought to purely reflect inactive brain states (e.g., Pfurtscheller et al. 1996). However, over the past decades, converging findings have challenged this perspective, suggesting that alpha oscillations may play an active role in shaping the timing of neural processing (Klimesch et al. 2007; VanRullen 2016; Di Gregorio et al. 2022; Wutz 2024; Schoffelen et al. 2024; Samaha and Romei 2024). Importantly, resting‐state alpha oscillatory activity has been shown to predict the excitability of the visual system in both healthy individuals (Klimesch et al. 2007; Sadaghiani and Kleinschmidt 2016) and clinical populations (Pietrelli et al. 2019; Allaman et al. 2021; Gallina, Pietrelli, et al. 2022; Gallina, Zanon, et al. 2022), ultimately suggesting that the alpha rhythm may reflect a neurophysiological marker of visual system functionality (Romei et al. 2008). Nevertheless, although alpha oscillations play a pivotal role, multiple neural rhythms coexist and collectively shape the functionality of the visual system (Ronconi and Melcher 2017; Ronconi et al. 2024). This is particularly evident in the case of theta oscillations (4–7 Hz), which are instrumental in organizing visuo‐attentional performance by orchestrating neural activity across distributed brain regions (Keller et al. 2017; Michel et al. 2022; Gallina et al. 2024). For instance, it has been suggested that during visual information processing, perceptual and attentional sampling mechanisms operate at distinct oscillatory rhythms, specifically within the alpha and theta bands, respectively (Michel et al. 2022; Gallina et al. 2024). This coordination is especially prominent in frontal and midline structures, where both resting‐state and task‐related theta activity have been linked to the top‐down regulation of attentional resources (Marek and Dosenbach 2018; Ahn et al. 2022; Gallina et al. 2024).

However, neural oscillatory activity is not a static feature of the neural system, and given its paramount role in shaping visual cortex excitability and perceptual performance, rhythmic stimulation techniques have been developed to functionally modulate this brain rhythm through a process commonly referred to as “entrainment” of neural oscillations (for recent reviews, see: Lakatos et al. 2019; Gallina et al. 2023; Duecker et al. 2024). Neural entrainment is defined as the temporal synchronization of endogenous neural activity to an external driving rhythm, leading to an alignment in phase and frequency and an enhancement in the power of ongoing neural oscillations (Lakatos et al. 2019). Based on this entrainment mechanism, studies using transcranial alternating current stimulation (tACS; Zaehle et al. 2010; Helfrich et al. 2014; Vossen et al. 2015; Borghini et al. 2018; Bender et al. 2019; Huang et al. 2021), transcranial magnetic stimulation (TMS; Thut et al. 2011; Coldea et al. 2022; Di Gregorio et al. 2022), and rhythmic sensory entrainment have consistently showed an effective synchronization of brain oscillations to the external stimulation frequency (for recent reviews, see: Lakatos et al. 2019; Gallina et al. 2023; Duecker et al. 2024).

In this realm, alpha‐band entrainment delivered through visual modality has proven to be a highly effective method for shaping occipito‐parietal oscillatory dynamics (Mathewson et al. 2011; Spaak et al. 2014; Ronconi et al. 2016; Kizuk and Mathewson 2017; Ronconi et al. 2018; Keitel et al. 2018, 2019; Wiesman and Wilson 2019; Gray and Emmanouil 2020; Fakche and Dugué 2024; Szaszkó et al. 2024). Administering short trains of alpha‐band visual rhythmic stimulation (~0.5–5 s) has consistently been shown to entrain frequency‐sensitive occipito‐parietal oscillations (Mathewson et al. 2012; Gray and Emmanouil 2020), suggesting that visual rhythmic stimulation may be applied to functionally model alpha neural activity (Gallina et al. 2023; Duecker et al. 2024). Importantly, the transient effects induced by alpha‐band sensory entrainment do not immediately return to baseline levels after the stimulation offset but instead persist for approximately 3–5 alpha cycles (~500 ms; e.g., Spaak et al. 2014; Wiesman and Wilson 2019). Similarly, visual entrainment has been implemented by embedding other stimulation rhythms (Gallina et al. 2023; Duecker et al. 2024). For example, theta‐band visual entrainment has primarily been applied trial‐by‐trial to modulate and investigate the role of theta oscillations in visual memory, sensory processing, and attentional mechanisms, by influencing fronto‐midline neural oscillatory circuits, leading to transient modulations in cognitive performance (Sato 2013; Wang et al. 2018; Roberts et al. 2018; Köster et al. 2019; Albouy et al. 2022; Ruikes et al. 2024; Venugopal et al. 2025).

However, despite its promising potential, some aspects of the effects of visual entrainment on neural oscillations remain under debate. First, whether prolonged visual entrainment can induce persistent modulations of neural oscillatory parameters remains unexplored. Given the importance of resting‐state alpha oscillations in balancing sensory cortices excitability (e.g., Romei et al. 2008), it is highly relevant to investigate whether prolonged alpha‐band sensory stimulation can elicit persistent modulations in resting‐state alpha oscillatory dynamics, potentially driving a functional reorganization of the underlying neural oscillatory networks that can persist well beyond the cessation of stimulation through mechanisms of neural plasticity (Neuling et al. 2013; Vossen et al. 2015; Kasten et al. 2016). Indeed, the nature of neural entrainment remains debated, particularly regarding whether the observed synchrony between neural activity and rhythmic stimulation reflects a modulation of intrinsic neural oscillatory dynamics, or can instead be explained by stimulus‐locked evoked responses and resonance phenomena (e.g., Capilla et al. 2011; Gallina et al. 2023; Duecker et al. 2024). This distinction is especially relevant when interpreting potential long‐lasting effects of rhythmic stimulation, as identifying neural signatures that extend beyond stimulus‐locked responses is critical for demonstrating reliable entrainment after‐effects. Accordingly, prolonged rhythmic stimulation may result either in a post‐entrainment change in oscillatory activity that perfectly matches the externally imposed rhythm, observable as a shift in neural oscillatory frequency, or in specific changes in intrinsic oscillatory dynamics, reflected in long‐lasting modulations of endogenous oscillatory power alongside increased coordination of oscillatory timing, consistent with theoretical accounts suggesting that rhythmic stimulation primarily interacts with endogenous neural oscillators rather than imposing externally driven rhythms (Helfrich et al. 2019; Duecker et al. 2024).

Relatedly, while maximal modulations occur when the stimulation frequency is tuned to the individual alpha frequency (IAF; Zaehle et al. 2010; Thut et al. 2011; Notbohm et al. 2016), it remains unclear whether deviations from the IAF could result in different oscillatory outcomes. Additionally, previous studies employing short‐term sensory entrainment have demonstrated that these effects are primarily localized in posterior brain areas contralateral to the stimulation (Thut et al. 2011; Spaak et al. 2014). However, it is still unexplored whether prolonged alpha‐band entrainment, unlike short‐term stimulation, may induce more widespread effects strengthening resting‐state alpha activity over different oscillatory circuits (Zhang et al. 2018; Alamia and VanRullen 2019, 2024). Similarly, visual entrainment at theta frequencies has typically been administered using short trains of stimulation with neural oscillatory effects observed in fronto‐central oscillatory networks dissipating within a few hundred milliseconds to several seconds after entrainment offset (Sato 2013; Wang et al. 2018; Roberts et al. 2018; Köster et al. 2019; Albouy et al. 2022; Ruikes et al. 2024; Venugopal et al. 2025), thereby leaving unresolved whether theta entrainment may induce persistent effects when prolongedly administered. Furthermore, given that alpha and theta oscillations have distinct topographical distributions and source generators (Schürmann and Başar 1994), visual entrainment administered at alpha and theta bands targets different, yet still interconnected, oscillatory circuits (Köster et al. 2019; Albouy et al. 2022). However, whether these different oscillatory networks exhibit similar sensitivity to visual entrainment and are capable of prolonged plasticity following rhythmic visual stimulation remains unclear.

To address these questions, in the current study we acquired EEG oscillatory signal before and after trains of prolonged visual entrainment (i.e., 1 min of duration) to investigate whether visual rhythmic stimulation, presented in the right or left hemifields (across different experimental sessions) at various frequencies within the alpha (IAF, IAF −2 Hz, and IAF +2 Hz) and theta (4.5 Hz) bands, could induce persistent effects on ongoing alpha and theta spectral measures (i.e., power, phase, and speed) immediately following the entrainment period, thus promoting potential long‐lasting plastic changes in underlying resting‐state oscillatory networks, which do not reflect a mere continuation of the externally driven rhythm, but rather sustained effects of rhythmic stimulation on endogenous neural systems (Helfrich et al. 2019; Duecker et al. 2024). This was achieved by comparing EEG eyes‐closed resting‐state oscillatory activity in alpha (i.e., 7–13 Hz) and theta (i.e., 3–6 Hz) bands before and after the entrainment stimulation. Importantly, we examined both the frequency specificity and topographic dependency of prolonged visual entrainment on neural oscillatory activity. Previous research has demonstrated that visual entrainment selectively modulates targeted oscillatory systems (e.g., Spaak et al. 2014; Keitel et al. 2018, 2019). Accordingly, we investigated whether alpha and theta entrainment specifically modulate oscillatory activity within the alpha and theta bands to ensure the frequency‐dependency specificity of sensory entrainment on neural oscillations. In terms of topographic specificity, we explored the scalp regions where the strongest effects of alpha and theta stimulation could be observed, further inspecting whether the prolonged entrainment effects might be diffuse rather than topographically focal, in contrast to short‐term entrainment, examining oscillatory activity across posterior, central, and anterior scalp areas. Additionally, with respect to alpha‐band entrainment, we assessed which alpha stimulation frequency (i.e., IAF, IAF −2 Hz, IAF +2 Hz) was most effective in eliciting maximal effects on post‐entrainment resting‐state activity.

## 2 Methods

### 2.1 Participants

A total of 25 healthy young adults (14 females; mean age = 22.8, SD = 2.74) were recruited among university students. Participants did not receive compensation or course credits. All were volunteers and presented normal or corrected‐to‐normal vision and hearing. Exclusion criteria were self‐reported neurological and attention disorders, epilepsy, and photosensitivity. The sample size was determined based on practical considerations and on previous studies investigating neural entrainment and oscillatory dynamics using EEG, which typically include comparable sample sizes (e.g., Capilla et al. 2011; De Graaf et al. 2013; Notbohm et al. 2016). Although an a priori power analysis was not conducted prior to data collection, we performed a post hoc power analysis using G*Power (Faul et al. 2007) focusing on the main within‐subject factor Condition (5 levels) in repeated‐measures ANOVAs, which represented the primary factor of interest in the study. The input parameters were as follows: effect size *f* = 0.25 (medium effect size), significance level *α* = 0.05, total sample size *N* = 25, number of groups = 1, and number of measurements = 5. Under these assumptions, the analysis indicated a statistical power of 1 − *β* = 0.89 for detecting the main effect of Condition. This suggests that the present sample size provided adequate sensitivity to detect medium‐sized effects in the primary analyses. Participants were informed about the procedure and the purpose of the study and gave written informed consent. The study was designed and performed in accordance with the ethical principles of the Declaration of Helsinki and was approved by the ethical committee of the Department of Psychology “Renzo Canestrari” of the University of Bologna (Prot. 42483).

### 2.2 Apparatus and Stimuli

Participants were seated in a soundproof room, positioned with a chin rest at a viewing distance of 57 cm in front of a 24‐in. LED monitor (Acer) with a resolution of 1080 × 980 pixels and a vertical refresh rate of 144 Hz. Throughout the experimental procedure, eye movements were tracked and recorded using a pan‐tilt ASL 6000 eye‐tracking system, operating at a sampling rate of 60 Hz. Electroencephalographic (EEG) data were continuously recorded during the experimental session using a BrainAmp DC amplifier (BrainProducts GmbH, Germany) and Ag/AgCl electrodes (Acticap Slim, BrainProducts GmbH, Germany) placed at 61 scalp locations (Fp1, AF3, AF7, F1, F3, F5, F7, FC1, FC3, FC5, FT7, FT9, C1, C3, C5, T7, CP1, CP3, CP5, TP7, TP9, P1, P3, P5, P7, PO3, PO7, O1, Fp2, AF4, AF8, F2, F4, F6, F8, FC2, FC4, FC6, FT8, FT10, C2, C4, C6, T8, CP2, CP4, CP6, TP8, TP10, P2, P4, P6, P8, PO4, PO8, O2, Fz, CPz, Pz, POz, Oz). AFz and Cz were used as the online reference and ground electrodes, respectively. EEG data were recorded with a band‐pass filter ranging from 0.01 to 100 Hz and a sampling rate of 1000 Hz, with electrode impedances maintained below 25 KΩ. All visual stimuli were presented against a middle‐gray background. The rhythmic visual stimulation (i.e., visual entrainment) consisted of flickering white squares (6 × 6 cm) presented 15 cm lateral to a central black fixation cross (0.5 × 0.5 cm). Flickering white square in each visual entrainment train was displayed for three refresh cycles (21 ms), with each square separated by varying durations of a blank screen, depending on the stimulation frequency (see next section). For each participant, visual entrainment was presented to the right or left hemifield relative to the central fixation cross in separate, randomized sessions (different days).

### 2.3 Experimental Procedure

**FIGURE 1:**
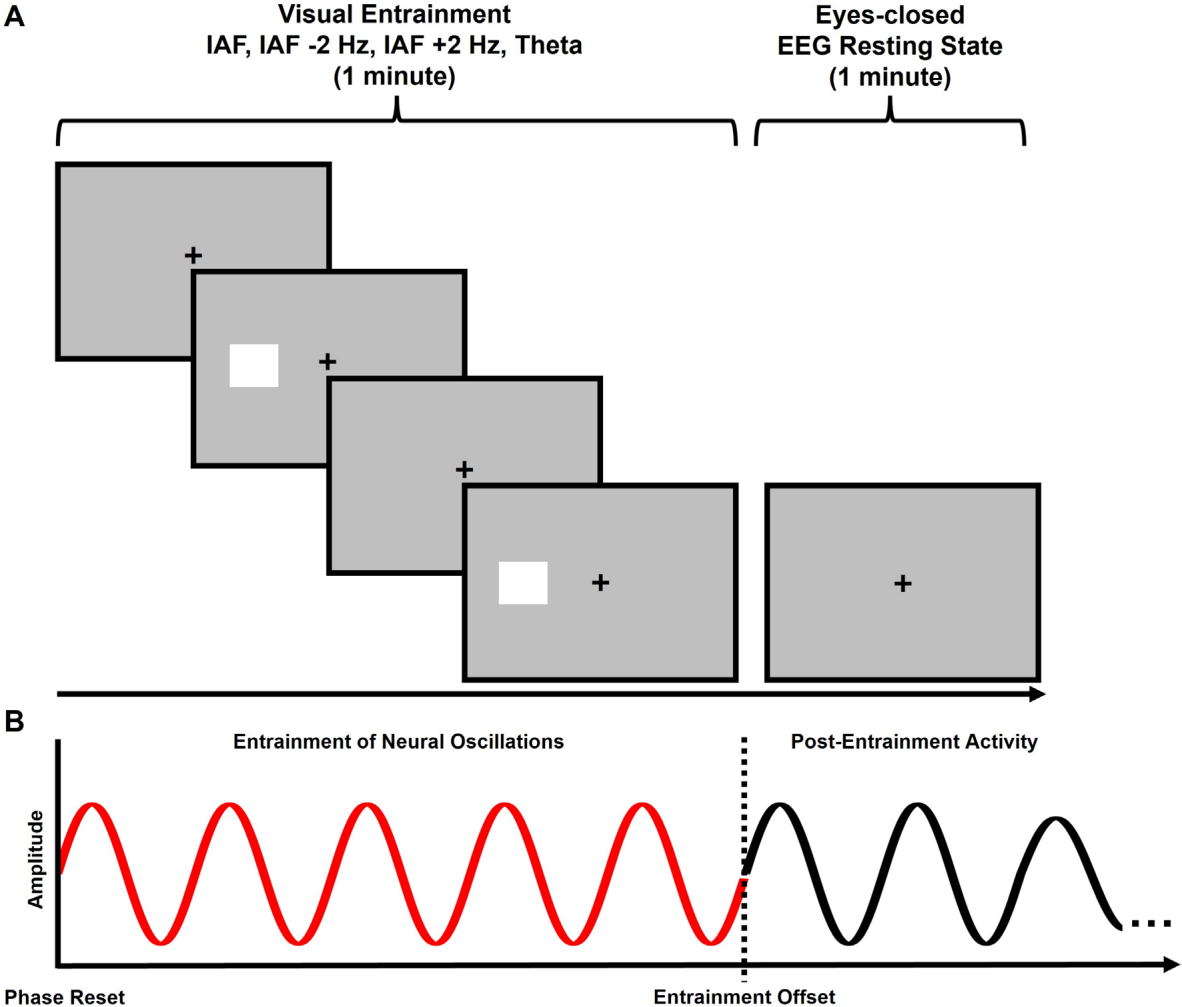
Schematic representation of the experimental paradigm. (A) The experiment began with the acquisition of baseline EEG signals during eyes‐closed resting state, comprising nine distinct 1‐min blocks. Following these baseline recordings, each participant's individual alpha frequency (IAF) was computed and visually examined to quantify baseline brain oscillatory activity and to establish the alpha‐band stimulation frequencies for subsequent visual entrainment. After determining the IAF, the visual entrainment protocol began. Specifically, participants were administered with visual entrainment at four different frequencies: IAF, two frequencies above (IAF +2 Hz) and below (IAF −2 Hz), and a stimulation condition within the theta range (4.5 Hz). Each condition (IAF, IAF +2 Hz, IAF −2 Hz, and theta) consisted of 1 min of visual entrainment followed by 1 min of eyes‐closed resting. This procedure (1 min of entrainment followed by 1 min of rest) was repeated nine times for each condition, resulting in nine entrainment and nine resting blocks per condition. Visual entrainment was applied separately to the right and left hemifields in randomized order across participants, with randomized stimulation sequences. Participants fixated on a central cross during throughout the experimental session. (B) Graphical representation depicting alpha‐band visual entrainment. A visual rhythmic stimulation administered at a specific frequency induces a phase reset of the endogenous brain oscillations in targeted brain areas, with a subsequent phase synchronization and an increase in amplitude. At the entrainment offset, the stimulation induced effects can persist for several cycles.

The experimental session (for a schematic representation, see Figure 1), began with the acquisition of baseline eyes‐closed resting‐state EEG signals, consisting of 9 separate 1‐min blocks. Following the baseline EEG recordings, each participant's individual alpha frequency (IAF) was calculated and visually inspected (for the details of the IAF processing and computation see the next section) to quantify their baseline brain oscillatory activity (in both alpha and theta bands) and to determine the alpha‐band stimulation frequencies for the subsequent visual entrainment. The computation of the IAF required approximately 3 min on average. Because the experiment consisted of two sessions conducted on different days, the IAF was recomputed independently at the beginning of each session. Importantly, no significant differences in IAF were observed between experimental sessions (see the section Entrainment‐induced effects on IAF for further details). After the computation of the IAF, the visual entrainment protocol started. In detail, participants underwent the visual entrainment protocol at four different frequencies: one corresponding to their IAF, two frequencies higher (IAF +2 Hz) and lower (IAF −2 Hz) relative to the IAF, and a stimulation condition within the theta band (4.5 Hz). Each entrainment protocol (IAF, IAF +2 Hz, IAF −2 Hz, and Theta) began with 1 min of visual entrainment, immediately followed by 1 min of eyes‐closed resting state. This design allowed for the assessment of the effects of visual entrainment on post‐entrainment eyes‐closed resting EEG activity compared to the baseline resting activity. This procedure (i.e., 1 min of entrainment followed by 1 min of eyes‐closed resting state) was repeated 9 times for each of the four stimulation conditions. During the visual entrainment phase, participants maintained their gaze on a central fixation cross and kept their eyes open in order to perceive the rhythmic visual flicker. Participants were trained before the beginning of the experiment to close their eyes immediately after the end of the stimulation period. Additionally, immediately after the end of each 1‐min entrainment train, a text instruction appeared on the screen indicating the start of the resting‐state period, and participants were instructed to close their eyes, remain relaxed, and avoid movements. Additionally, the experimenter ensured that these instructions were strictly followed and that all participants complied with the task requirements across all trials. The same eyes‐closed condition was used during the baseline resting‐state recordings, ensuring that pre‐ and post‐stimulation measurements were obtained under identical sensory conditions. This approach allowed the entrainment stimulation to be delivered under eyes‐open conditions while the resulting modulation of oscillatory activity was assessed during eyes‐closed resting‐state periods, minimizing ongoing visual input and facilitating the measurement of intrinsic oscillatory activity, particularly with the aim of identifying modulations of alpha‐band activity, which is known to be strongly influenced by visual input and typically more pronounced during eyes‐closed resting conditions (Klimesch 1999). The visual entrainment protocol was administered to the right and left hemifields in separate experimental sessions conducted in different days, with the order of presentation randomized across participants. Similarly, in each experimental session, the sequence of stimulation conditions (9 consecutive 1‐min entrainment trains for each stimulation condition) was randomized. Participants were instructed to maintain their gaze on a central fixation cross during the visual entrainment and to keep their head positioned on the chin rest throughout the entire experimental session.

### 2.4 Preprocessing and Data Analysis

#### 2.4.1 Individual Alpha Frequency Computation

The EEG signals recorded during the baseline condition were pre‐processed and analyzed offline using EEGlab (v.2022; Delorme and Makeig 2004) and custom scripts developed in Matlab (R2022a; The Mathworks Inc., USA). Data from all electrodes were re‐referenced to the average of all scalp electrodes and filtered with a band‐pass filter ranging from 1 to 100 Hz. Continuous EEG data were then segmented into 1‐s epochs. Dimensionality reduction was performed using Principal Component Analysis (PCA), reducing the data to 32 components. Independent Component Analysis (ICA) was subsequently applied, and components corresponding to horizontal and vertical eye movement artifacts were visually identified and removed. Subsequently, a Fast Fourier Transform (FFT) was computed on the artifact‐free EEG data, with a frequency resolution of 0.5 Hz. To determine the appropriate stimulation frequencies for the alpha‐band entrainment conditions (i.e., IAF, IAF +2 Hz, IAF −2 Hz), the individual alpha frequency (IAF) for each participant was estimated from parieto‐occipital regions of interest (ROIs) defined over the left hemisphere (P1, P3, P5, P7, PO3, PO7, O1) and the right hemisphere (P2, P4, P6, P8, PO4, PO8, O2). The IAF was computed from the average spectral activity across both posterior ROIs, independently of the stimulated hemifield. No significant differences in IAF were observed between the left and right posterior ROIs (see Results), supporting the use of the averaged posterior signal for determining stimulation frequencies. Based on the computation of the IAF, participants were stimulated at the following mean frequencies: IAF (10.39 ± 0.57 Hz), IAF +2 Hz (12.67 ± 0.58 Hz), and IAF −2 Hz (8.47 ± 0.57 Hz), while the theta stimulation frequency was fixed at 4.5 Hz.

#### 2.4.2 Analysis of Alpha‐ and Theta‐Band Activity: Power, Inter‐Trial Phase Coherence, and IAF

The EEG signals from all electrodes, acquired during the baseline condition and following the four entrainment conditions (IAF, IAF +2 Hz, IAF −2 Hz, and Theta), were re‐referenced to the average of all scalp electrodes and filtered with a 1–100 Hz band‐pass filter. The EEG data, consisting of nine separate 1‐min blocks of eyes‐closed resting‐state recordings, were combined and segmented into 1‐s epochs, resulting in 540 epochs for each experimental condition. Dimensionality was reduced to 32 components using Principal Component Analysis (PCA), and Independent Component Analysis (ICA) was applied to identify and remove components corresponding to horizontal and vertical eye artifacts. A Fast Fourier Transform (FFT) was performed on each of the 540 artifact‐free epochs, with a frequency resolution of 0.5 Hz. IAF and alpha power were calculated for each epoch as the peak and mean power (in dB), respectively, within the 7–13 Hz range for each electrode. Additionally, inter‐trial phase coherence (ITPC), a neural oscillatory measure reflecting evoked brain responses to rhythmic stimuli and the coordination of oscillatory timing (Spaak et al. 2014; Keitel et al. 2018, 2019; Gray and Emmanouil 2020), was computed. These measures allowed us to assess complementary signatures of entrainment, including potential shifts in oscillatory frequency, modulations of endogenous oscillatory power, and changes in phase consistency, thereby enabling us to distinguish between potential long‐lasting changes in intrinsic neural dynamics and effects attributable to stimulus‐locked evoked responses and resonance phenomena (Capilla et al. 2011; Duecker et al. 2024). Theta power and ITPC were calculated in the 3–6 Hz range using the same procedure. Power, ITPC and IAF values for each epoch were averaged across blocks, yielding 60 epochs for each experimental condition. To evaluate the duration of entrainment effects on subsequent 1‐min resting‐state activity, the averaged 60 epochs were divided into three segments, each containing 20 epochs. For statistical analysis, separate repeated measures ANOVAs were conducted to assess whether prolonged visual entrainment induced persistent modulations with widespread, rather than localized, effects on neural oscillatory activity. Two posterior parieto‐occipital regions of interest (ROIs)—in the left hemisphere (P1, P3, P5, P7, PO3, PO7, O1) and the right hemisphere (P2, P4, P6, P8, PO4, PO8, O2)—, two central ROIs—in the left hemisphere (CP5, C5, FC5, Cp3, C3, FC3, Cp1, C1, FC1) and right hemisphere (CP6, C6, FC6, Cp4, C4, FC4, Cp2, C2, FC2)—, and one anterior ROI (Fp1, Fp2, AF3, AF4, AF7, AF8, F1, F2, Fz, AFz) were selected. To examine differential effects of the four entrainment conditions on resting EEG activity in the posterior and central ROIs with respect to baseline, two separate repeated measures ANOVAs were performed on alpha and theta measures (i.e., power, ITPC, IAF) with factors including Condition (Baseline, post‐IAF, post‐IAF +2 Hz, post‐IAF −2 Hz, Theta), Temporal Segment (1st segment: 0–20 s; 2nd segment: 20–40 s; 3rd segment: 40–60 s), Hemisphere (Left and Right ROIs), and Stimulated Hemifield (Left or Right). Similarly, separate ANOVAs were conducted for the anterior ROI, with factors including Condition, Temporal Segment, and Hemifield Stimulated, for both alpha and theta measures. The Greenhouse–Geisser correction was applied in cases where the sphericity assumption was violated. All post hoc comparisons were performed with Tukey HSD test.

## 3 Results

### 3.1 Entrainment‐Induced Effects on Oscillatory Power

#### 3.1.1 Alpha‐Band (7–13 Hz) Power

**FIGURE 2:**
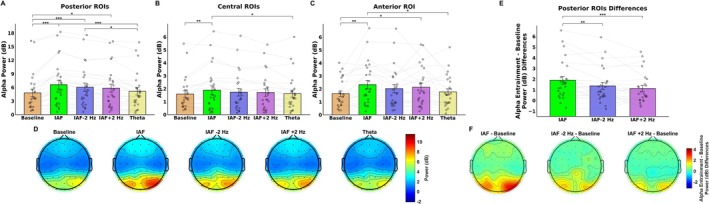
The figure highlights alpha‐band (7–13 Hz) power results following baseline and the different entrainment conditions. (A) Bar plot showing the effect of stimulation condition on posterior region of interests (ROIs). The results revealed that entrainment increase alpha power following alpha‐band (IAF, IAF −2 Hz, IAF +2 Hz), but not theta stimulation, as compared to baseline condition. (B) Bar plot showing the effect of stimulation condition on central ROIs. The results revealed that entrainment increase alpha power following IAF as compared to baseline and theta conditions. (C) Bar plot showing the effect of stimulation condition on anterior ROI. The results revealed that entrainment increase alpha power following IAF and IAF +2 Hz, but not IAF −2 Hz and theta stimulations, as compared to baseline condition. (D) Topographic scalp maps depicting alpha power over the scalp for each stimulation condition. (E) Bar plot showing the power differences observed between the post‐entrainment conditions in the alpha‐band and baseline activity. The results showed that entrainment delivered at the participants' IAF can induces stronger power modulations as compared to other alpha frequencies (i.e., IAF −2 Hz, IAF +2 Hz). (F) Topographic scalp maps depicting alpha power differences between each alpha‐band entrainment condition and baseline power. The error bars indicate the standard error of the mean (SEM); black circles show the individual values. *p < 0.05. **p < 0.01. ***p < 0.001.

For the posterior ROIs, the ANOVA performed on alpha power revealed significant main effects of Temporal Segment (*F*(2, 48) = 12.35, *p* < 0.001), Hemisphere (*F*(1, 24) = 4.81, *p* = 0.038), and Condition (*F*(4,96) = 15.84, *p* < 0.001), but no significant effect of Stimulated Hemifield (*F*(1, 24) = 0.19, *p* = 0.66). Post hoc comparisons for Temporal Segment indicated that alpha power was higher in the first segment (M = 6.20 dB, SD = 4.58) compared to the second (M = 5.76 dB, SD = 4.36; *p* = 0.036) and third (M = 5.35 dB, SD = 4.07; *p* < 0.001) segments, with a further decrease in alpha power observed from the second to the third segment (*p* = 0.049). Post hoc comparisons for Hemisphere showed that alpha power was higher in the right hemisphere (M = 6.25 dB, SD = 4.82) with respect to the left (M = 5.30 dB, SD = 4.25; *p* = 0.038). Regarding Condition main factor (Figure 2), post hoc comparisons revealed that alpha power was significantly higher following IAF (M = 6.69 dB; SD = 5.14; *p* < 0.001), IAF −2 Hz (M = 6.16 dB, SD = 4.89; *p* < 0.001), and IAF +2 Hz (M = 5.92 dB, SD = 4.66; *p* = 0.045), as compared to the baseline condition (M = 4.8 dB, SD = 4.07). However, alpha power following Theta stimulation (M = 5.29 dB, SD = 4.58) did not significantly differ from baseline (*p* = 0.34), indicating that only IAF‐range entrainment effectively increased alpha power. In addition, Theta condition showed a lower alpha power with respect to IAF (*p* < 0.001) and IAF −2 Hz (*p* = 0.011) conditions. Interestingly, no significant interactions were found between the main factors (*p* > 0.42), suggesting that the effects induced by the alpha‐band entrainment on alpha power were independent of the temporal segment, hemisphere, and stimulated hemifield. Consequently, these effects appeared to be sustained over time and topographically distributed both ipsilaterally and contralaterally to the stimulated hemifield. Importantly, these effects were also consistent across the two experimental sessions. Specifically, the absence of an interaction between Condition and Stimulated Hemifield (corresponding to the two experimental sessions administered on separate days) indicates that the magnitude of the entrainment effects did not differ between sessions, suggesting that the observed modulations of resting‐state oscillatory activity were stable across experimental days.

To further investigate differential effects elicited by the different stimulation frequencies administered within the alpha‐band (i.e., IAF, IAF −2 Hz, IAF +2 Hz), we calculated the power differences observed between the post‐entrainment conditions and baseline activity (Figure 2E,F). Subsequently, we performed an additional repeated measures ANOVA on these power differences, with factors including Condition Differences (i.e., IAF—Baseline, IAF −2 Hz—Baseline, IAF +2 Hz—Baseline), Temporal Segment, Hemisphere, and Stimulated Hemifield. This analysis revealed a significant main effect of Condition Differences (*F*(2, 48) = 11.16, *p* < 0.001; Figure 2E,F) indicating stronger entrainment modulations (i.e., greater power differences between baseline and post‐entrainment activity) following IAF stimulation (M = 1.89, SD = 1.08) compared to both the IAF −2 Hz (M = 1.36, SD = 0.89; *p* = 0.007) and IAF +2 Hz conditions (M = 1.11, SD = 0.96; *p* < 0.001). No significant power differences were observed between the IAF −2 Hz and IAF +2 Hz conditions (*p* = 0.31). These findings align with previous evidence suggesting that rhythmic stimulation at the participants' IAF can induce stronger resonance phenomena in neural oscillatory activity (Regan 1982; Pikovsky et al. 2003; Thut and Gross 2012).

For the central ROIs, the ANOVA performed on the alpha power did not reveal a significant main effect of Stimulated Hemifield (*F*(1, 24) = 0.167, *p* = 0.68), but significant main effects of Temporal Segment (*F*(2, 48) = 9.08, *p* < 0.001) and Condition (*F*(4, 96) = 3.30, *p* = 0.013; Figure 2B). Post hoc comparisons for the Temporal Segment indicated that alpha power was higher in the first segment (M = 1.83 dB, SD = 0.88) compared to the third segment (M = 1.57 dB, SD = 0.93; *p* < 0.001). Regarding Condition main factor, post hoc comparisons revealed that alpha power was significantly increased following IAF (M = 1.88 dB; SD = 0.96) as compared to baseline (M = 1.59 dB; SD = 0.92; *p* = 0.012) and theta (M = 1.62 dB; SD = 0.74; *p* = 0.032) conditions.

For the anterior ROI, the ANOVA performed on the alpha power did not reveal a significant main effect of Stimulated Hemifield (*F*(1, 24) = 0.45, *p* = 0.5), but significant main effects of Temporal Segment (*F*(2, 48) = 5.77, *p* = 0.005) and Condition (*F*(4, 96) = 5.20, *p* < 0.001; Figure 2C). Post hoc comparisons for Temporal Segment indicated that alpha power was higher in the first segment (M = 2.17 dB, SD = 1.23) compared to the third segment (M = 2.0 dB, SD = 1.33; *p* = 0.004). Regarding Condition main factor, post hoc comparisons revealed that alpha power was significantly increased following IAF (M = 2.39 dB; SD = 1.33; *p* = 0.003) and IAF + 2 Hz (M = 2.3 dB, SD = 2.28; *p* = 0.019) with respect to the baseline condition (M = 1.76 dB, SD = 1.13), with a further significant power difference between IAF and Theta stimulation condition (M = 1.85 dB, SD = 1.27; *p* = 0.016). Furthermore, alpha power following Theta entrainment did not significantly differ from baseline (*p* = 0.98), suggesting that only IAF and IAF + 2 Hz stimulation modulated alpha power. Importantly, consistent with the findings in the posterior ROIs, the ANOVA revealed no significant interactions between the main factors (p‐values > 0.107), indicating that the modulations of alpha power induced by the entrainment do not vary across temporal segments or the stimulated hemifield.

#### 3.1.2 Theta‐Band (3–6 Hz) Power

In contrast to the findings for alpha power, the control ANOVA conducted on theta power over the posterior ROIs revealed a significant main effect of Hemisphere (*F*(1, 24) = 5.91, *p* = 0.022), with higher theta power observed in the right hemisphere (M = 0.76 dB, SD = 0.034) compared to the left hemisphere (M = 0.72, SD = 0.41; *p* = 0.013). No other significant main effects or interactions were found (*p* > 0.117).

**FIGURE 3:**
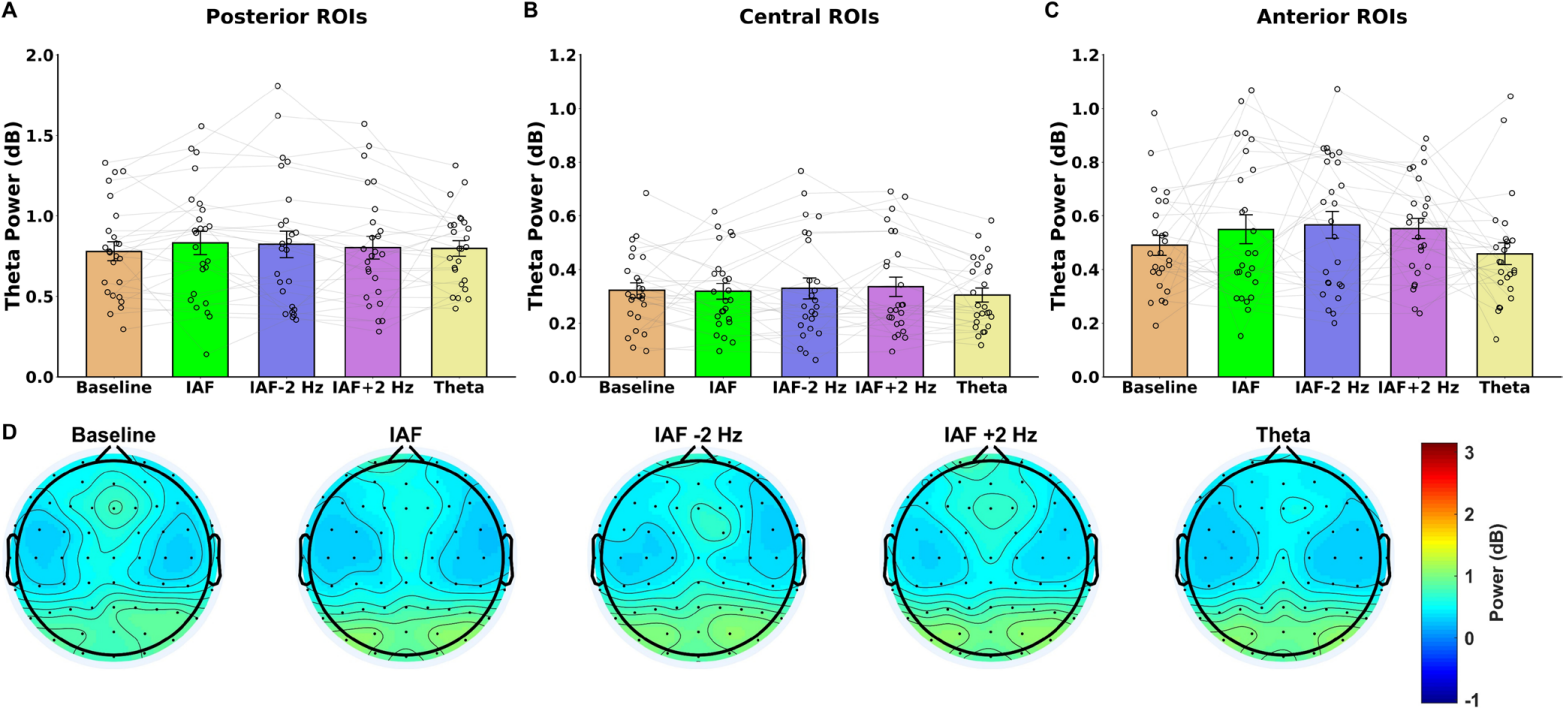
The figure highlights theta‐band (3–6 Hz) power results following baseline and the different entrainment conditions. Bar plot showing the effect of stimulation condition on posterior region of interests (ROIs; A), central (B) and anterior ROIs (C), revealing no entrainment‐induced modulations on theta power. (D) Topographic scalp maps depicting theta power over the scalp for each stimulation condition. The error bars indicate the standard error of the mean (SEM); black circles show the individual values.

For the central ROIs, the ANOVA revealed a significant main effect of Hemisphere (*F*(1, 24) = 6.04, *p* = 0.021), with higher theta power observed in the right hemisphere (M = 0.34 dB, SD = 0.36) compared to the left hemisphere (M = 0.302, SD = 0.41; *p* = 0.021). Similarly, in the anterior ROI, no significant main effects or interactions were observed (*p* > 0.136). These results suggest that the entrainment administered within the alpha and theta bands did not induce any modulation of theta power both in posterior and anterior ROIs (Figure 3).

### 3.2 Entrainment‐Induced Effects on Oscillatory ITPC

#### 3.2.1 Alpha‐Band (7–13 Hz) ITPC

**FIGURE 4:**
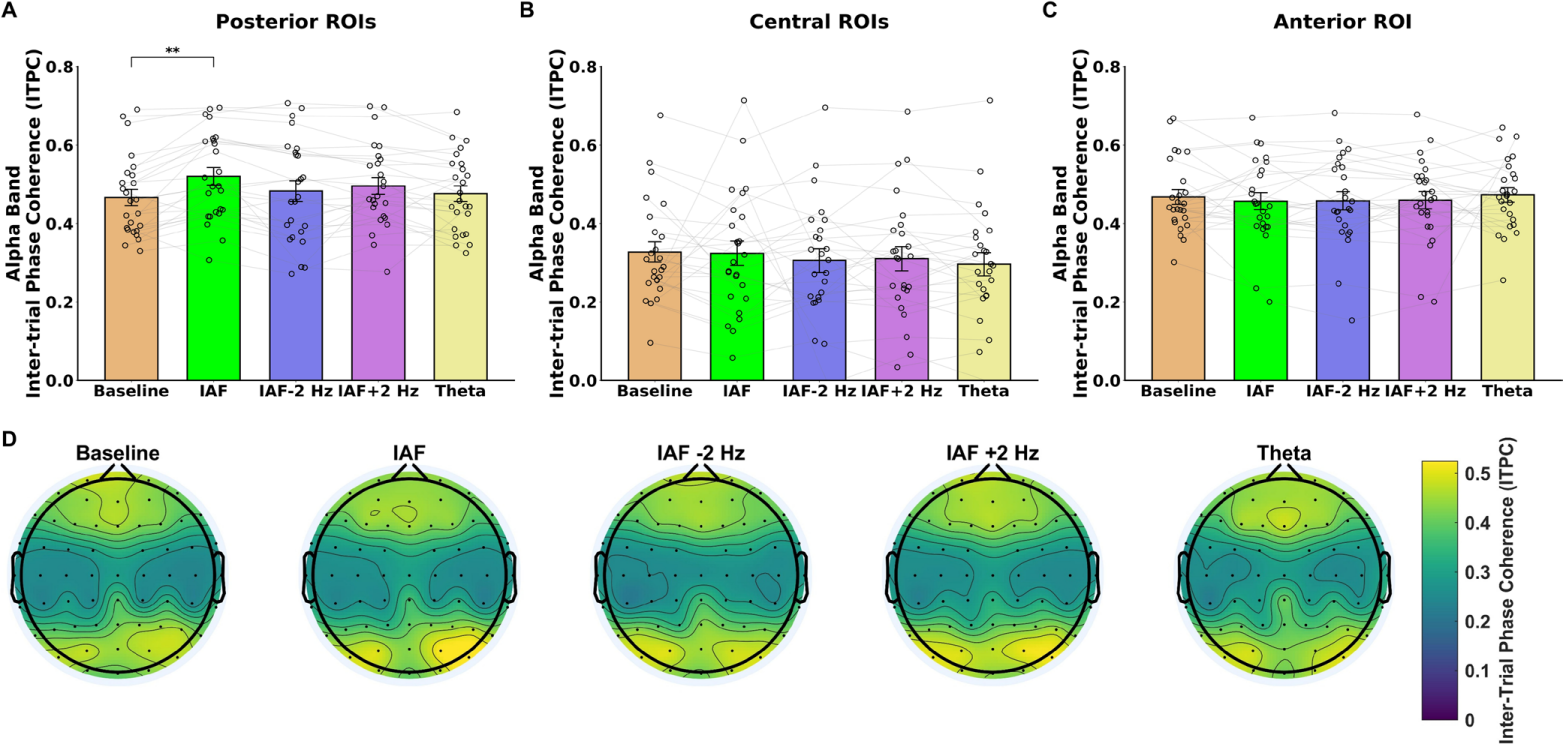
The figure highlights alpha‐band (7–13 Hz) Inter‐Trial Phase Coherence (ITPC) results following baseline and the different entrainment conditions. Bar plots showing the effect of stimulation condition on posterior (A), central (B) and anterior ROIs (C). The results revealed that entrainment increases alpha power following IAF entrainment over the posterior scalp areas with respect to baseline condition. (D) Topographic scalp maps depicting alpha ITPC over the scalp for each stimulation condition. The error bars indicate the standard error of the mean (SEM); black circles show the individual values. **p < 0.01.

For the posterior ROIs, the ANOVA performed on alpha ITPC revealed significant main effects of Temporal Segment (*F*(2, 48) = 4.79, *p* = 0.012) and Condition (*F*(4,96) = 3.51, *p* = 0.01), but no significant effect of Stimulated Hemifield (*F*(1, 24) = 0.72, *p* = 0.4) and Hemisphere (F(1, 24) = 0.95, *p* = 0.33). Post hoc comparisons for Temporal Segment indicated that alpha ITPC was higher in the first segment (M = 0.5 dB, SD = 0.14) compared to the third segment (M = 0.47 dB, SD = 0.16; *p* = 0.01); all the other comparisons were not significant (*p* > 0.11). Importantly, post hoc comparisons of the Condition factor revealed significantly higher ITPC following IAF stimulation (M = 0.52, SD = 0.16; Figure 4A) compared to the baseline condition (M = 0.46, SD = 0.12; *p* = 0.004), with no other significant comparisons (*p* > 0.09), indicating that only IAF entrainment increased alpha ITPC. No significant interactions were found between the main factors (*p* > 0.07), suggesting that the effects of IAF stimulation on alpha ITPC were consistent across temporal segments, hemispheres, and stimulated hemifield, and were therefore sustained over time and topographically distributed both ipsilaterally and contralaterally to the stimulated hemifield.

For the central ROI, the ANOVA performed on alpha ITPC revealed significant main effects of Temporal Segment (*F*(2, 48) = 11.19, *p* < 0.001), but not other main effects or interactions (*p* > 0.055). Post hoc comparisons for Temporal Segment indicated that alpha ITPC was lower in the first segment (M = 0.29 dB, SD = 0.11) compared to the second (M = 0.317 dB, SD = 0.21; *p* = 0.0018) and third segment (M = 0.321 dB, SD = 0.19; *p* < 0.001).

On the other hand, in the anterior ROI, the ANOVA conducted on alpha ITPC revealed no significant main effects or interactions (*p* > 0.067; Figure 4C), indicating that none of the entrainment conditions modulated ITPC in the anterior scalp region.

#### 3.2.2 Theta‐Band (3–6 Hz) ITPC

**FIGURE 5:**
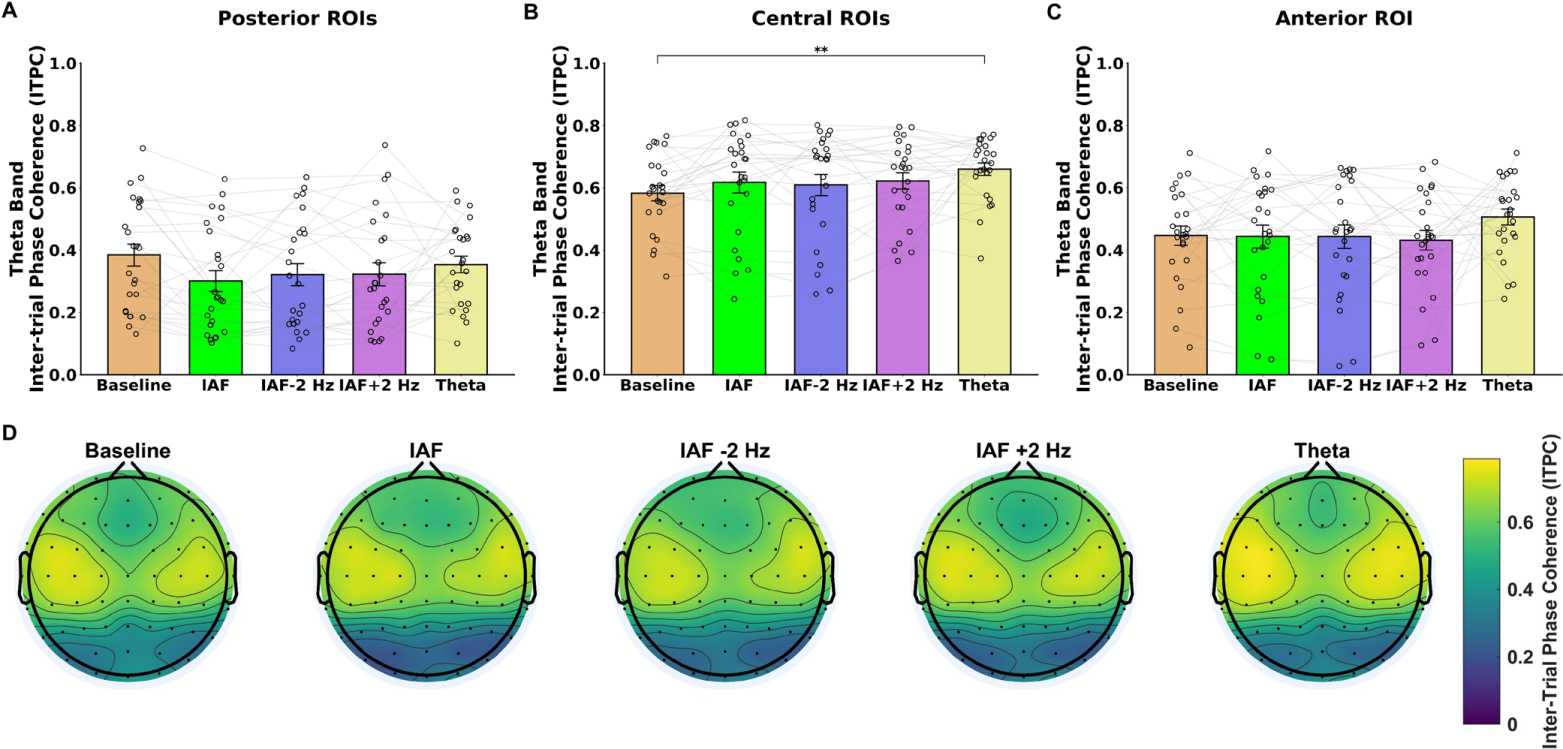
The figure highlights theta‐band (3–6 Hz) Inter‐Trial Phase Coherence (ITPC) results following baseline and the different entrainment conditions. (A) Bar plot showing the effect of stimulation condition on posterior region of interests (ROIs). The results did not reveal any entrainment‐induced modulations on theta ITPC over the posterior ROIs. (B) Bar plot showing the effect of stimulation condition on anterior ROI. The results revealed a significant ITPC modulation following theta stimulation as compared to the baseline condition. (C) Bar plot showing the effect of stimulation condition on anterior ROI. The results did not show significant power modulations. (D) Topographic scalp maps depicting theta ITPC over the scalp for each stimulation conditions. The error bars indicate the standard error of the mean (SEM); black circles show the individual values. **p < 0.01.

The ANOVA performed on theta ITPC showed a significant main effect of Stimulated Hemifield (*F*(1, 24) = 4.81, *p* = 0.038) and Condition (*F*(4, 96) = 2.72, *p* = 0.033), but no significant effect of Hemisphere (*F*(1, 24) = 3.20, *p* = 0.086) and Temporal Segment (*F*(2, 48) = 2.77, *p* = 0.072). Post hoc comparisons of Stimulated Hemifield revealed a higher theta ITPC following stimulation delivered in the right hemifield (M = 0.39, SD = 0.13) as compared to the left one (M = 0.34, SD = 0.18; *p* = 0.038). Regarding the Condition main effect (Figure 5A), such analysis did not show significant post hoc comparison among stimulation conditions (*p* > 0.08). No significant interactions were found between the main factors (*p* > 0.058).

For the central ROI, the ANOVA performed on the theta ITPC revealed a significant main effect of Hemisphere (*F*(1, 24) = 10.92, *p* = 0.002), Temporal Segment (*F*(2, 48) = 9.12, *p* < 0.001), and Condition (*F*(4, 96) = 3.41, *p* = 0.011). Post hoc comparisons of Hemisphere revealed a higher theta ITPC in the left hemisphere (M = 0.654, SD = 0.26) as compared to the right one (M = 0.628, SD = 0.34; *p* = 0.031). Post hoc comparisons of Temporal Segment revealed a higher theta ITPC in the first Temporal Segment (M = 0.651, SD = 0.44) as compared to the second (M = 0.636, SD = 0.51; *p* = 0.001) and third one (M = 0.637, SD = 0.29; *p* = 0.002). Regarding the Condition main effect (Figure 5A), post hoc comparisons revealed a theta ITPC increase following theta entrainment (M = 0.697, SD = 0.41) with respect to the ITPC observed during the baseline condition (M = 0.602, SD = 0.36; *p* = 0.005).

For the anterior ROI, the ANOVA performed on the theta ITPC did not reveal a significant main effect of Stimulated Hemifield (*F*(1, 24) = 0.99, *p* = 0.32) and Condition (*F*(4, 96) = 1.33, *p* = 0.26), but a significant main effect of Temporal Segment (*F*(2, 48) = 3.55, *p* = 0.036). Post hoc comparisons indicated that theta ITPC was higher in the first segment (M = 0.72, SD = 0.24) compared to the second segment (M = 0.7, SD = 0.21; *p* = 0.037). All the other comparisons, including the Condition factor (Figure 5B), were not significant (p‐values > 0.13). No significant interactions were found between the main factors (*p* > 0.51).

### 3.3 Consistency and Stability of Entrainment Effects

Although the main analyses revealed consistent effects of visual entrainment on oscillatory power and ITPC, suggesting persistent modulations of the neural oscillatory system, additional analyses were conducted to ensure that these effects were not driven by potential confounds. First, we examined whether the observed entrainment‐induced modulations reflected transient responses immediately following rhythmic stimulation, rather than sustained entrainment‐related activity. To this aim, we repeated all analyses on oscillatory power and ITPC after excluding the first three epochs of the first temporal segment (corresponding to the first 3000 ms of the post‐stimulation period). This procedure ensured that any potential transient evoked responses associated with the final stimuli of the entrainment train were removed from the analyses. The results of these analyses replicated the pattern of findings observed in the main analyses (for further details, see Results S1). Specifically, the significant effects of Condition on oscillatory power and ITPC remained unchanged across ROIs and frequency bands when the initial post‐stimulation interval was excluded (see Results S1 and Table S1). These results indicate that the observed modulations were not driven by transient activity immediately following stimulation offset. To further examine the temporal dynamics of these effects, we analyzed the second‐by‐second evolution of oscillatory power and ITPC across the post‐stimulation period. As illustrated in Figure S1, the entrainment‐induced modulations persisted across the entire post‐stimulation interval rather than decaying rapidly within the first few seconds after stimulation. Together, these analyses indicate that the observed changes in oscillatory activity reflect sustained entrainment‐related modulations of resting‐state neural dynamics rather than brief transient responses following rhythmic stimulation.

Furthermore, to assess whether the entrainment effects were stable across the experimental session, we conducted additional control analyses examining potential time‐on‐task effects across the nine post‐stimulation resting‐state blocks. Indeed, oscillatory activity is known to fluctuate over time as a function of vigilance and fatigue, often accompanied by increases in low‐frequency oscillatory activity such as alpha and theta power (e.g., Benwell et al. 2019; Arnau et al. 2021). The consistency of the entrainment effects over time was therefore evaluated by testing whether the magnitude of the observed modulations varied across the nine resting‐state blocks. To this aim, repeated‐measures ANOVAs were performed separately for power and ITPC measures and separately for each ROI, including Condition (Baseline, IAF, IAF −2 Hz, IAF +2 Hz, Theta) and Resting‐State Block (nine levels) as within‐subject factors. Overall, the analyses replicated the previously reported main effects of Condition (see previous sections), while no significant main effect of Resting‐State Block and no Condition × Resting‐State Block interaction were observed across ROIs and oscillatory measures (all *p* > 0.05), indicating that the magnitude of the entrainment effects remained stable across the nine resting‐state blocks (for a detailed report of these analyses, see Table S2 and Figure S2). The only exception was observed for theta power in the anterior ROI, where a significant main effect of Resting‐State Block emerged (*F*(8,192) = 3.41, *p* = 0.005). Post hoc comparisons revealed a significant increase in theta power between resting‐state block 1 and block 9 (*t* = −0.608, *p* = 0.0179), whereas the remaining comparisons did not reach statistical significance, thus revealing a late influence of time on task and/or fatigue on theta power, evident only during the last block of resting state. Importantly, no Resting‐State Block × Condition interaction was found (*F*(32,768) = 1.21, *p* = 0.19), indicating that this increase occurred similarly across stimulation conditions. Overall, these control analyses indicate that the entrainment‐induced modulations of resting‐state oscillatory activity remained stable across the experimental session and were not driven by progressive time‐on‐task changes in neural oscillatory dynamics.

### 3.4 Entrainment‐Induced Effects on IAF

**FIGURE 6:**
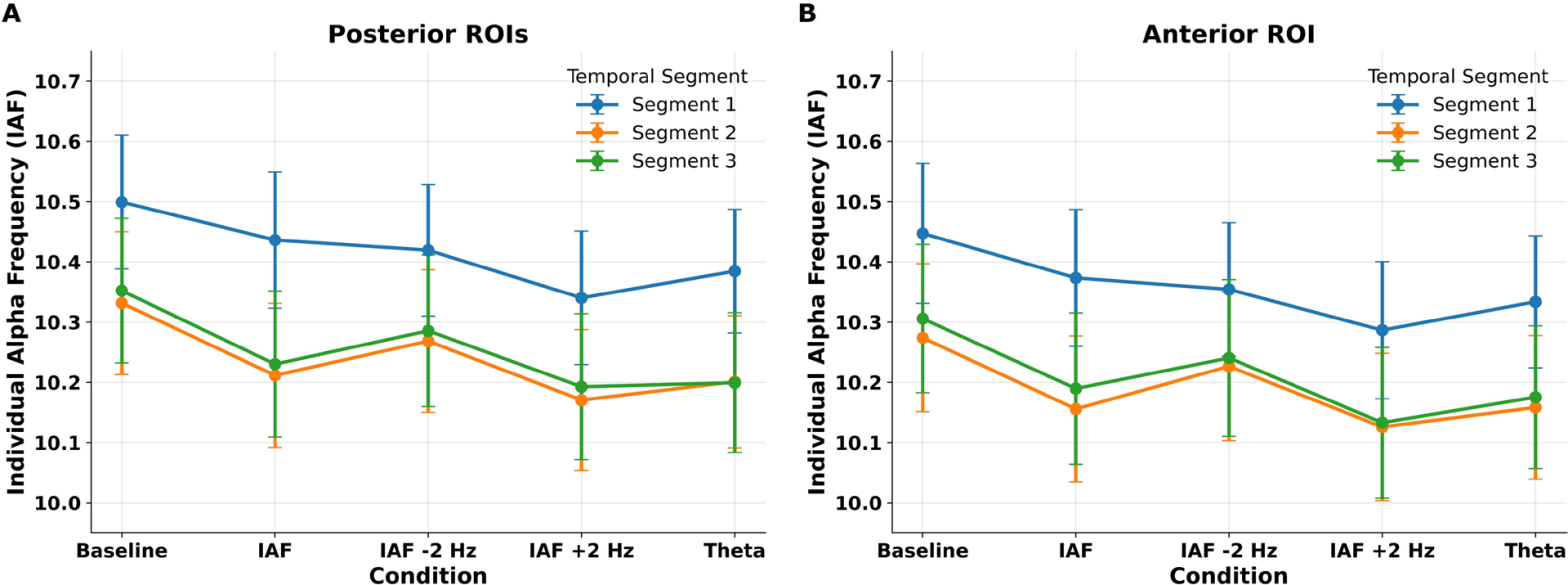
The figure illustrates the effects on peak Individual Alpha Frequency (IAF) as a function of Condition (Baseline, IAF, IAF –2 Hz, IAF +2 Hz, Theta) and Temporal Segment (Segment 1 = blue, Segment 2 = orange, Segment 3 = green), separately for the posterior region of interest (ROI; Panel A) and the anterior ROI (Panel B). The error bars indicate the standard error of the mean (SEM).

We also analyzed potential shifts in the frequency peak (separately identified as the highest peak in both power spectrum—i.e., IAF—and ITPC) induced by the different stimulation frequencies within the alpha‐band (i.e., IAF, IAF +2 Hz, and IAF −2 Hz) in comparison with each other and the baseline condition (Figure 6).

The ANOVA conducted on IAF observed in the power spectrum over the posterior ROIs revealed a significant main effect of Temporal Segment (*F*(2, 48) = 29.96, *p* < 0.001). No other significant main effects or interactions were found (*p* > 0.12). Temporal Segment post hoc comparisons showed a faster IAF in the first segment (M = 10.41 Hz, SD = 0.81) compared to the second (M = 10.23 Hz, SD = 0.77; *p* < 0.001) and third (M = 10.25 dB, SD = 0.68; *p* < 0.001) segments, suggesting that, regardless of stimulation conditions, stimulated hemifield and hemisphere, the IAF speed decreases over time. All the other comparisons were not significant (*p* > 0.13). Similarly, the ANOVA performed on anterior ROI showed a significant main effect of Temporal Segment (*F*(2, 48) = 37.84, *p* < 0.001), indicating a faster IAF in the first segment (M = 10.18 Hz, SD = 0.89) compared to the second (M = 10.35 Hz, SD = 1.11; *p* < 0.001) and third (M = 10.20 dB, SD = 0.97; *p* < 0.001) segments. No other significant main effects or interactions were found (*p* > 0.14).

The ANOVA conducted on the frequency peak observed in the ITPC over the posterior ROIs revealed no significant main effects or interactions (*p* > 0.063). Similarly, the ANOVA on anterior ROI did not show significant main effects or interactions (*p* > 0.19).

Overall, these results suggest that, although a relative decrease in IAF speed was observed over time, none of the entrainment conditions induced a speeding up or slowing down of the frequency peak.

### 3.5 Correlation Between Baseline and Post‐Entrainment Alpha Power and ITPC

Based on the significant modulations of alpha power and ITPC induced by entrainment administered within the alpha‐band, we hypothesized that baseline oscillatory activity might influence the strength of the observed post‐entrainment activity modulations, as previously reported (Helfrich et al. 2014). Accordingly, we performed Pearson correlational analyses separately for alpha power and ITPC measures, and separately for posterior and anterior ROIs, linking baseline activity to the differences observed between the different entrainment conditions (i.e., IAF, IAF −2 Hz and IAF +2 Hz) and baseline.

**FIGURE 7:**
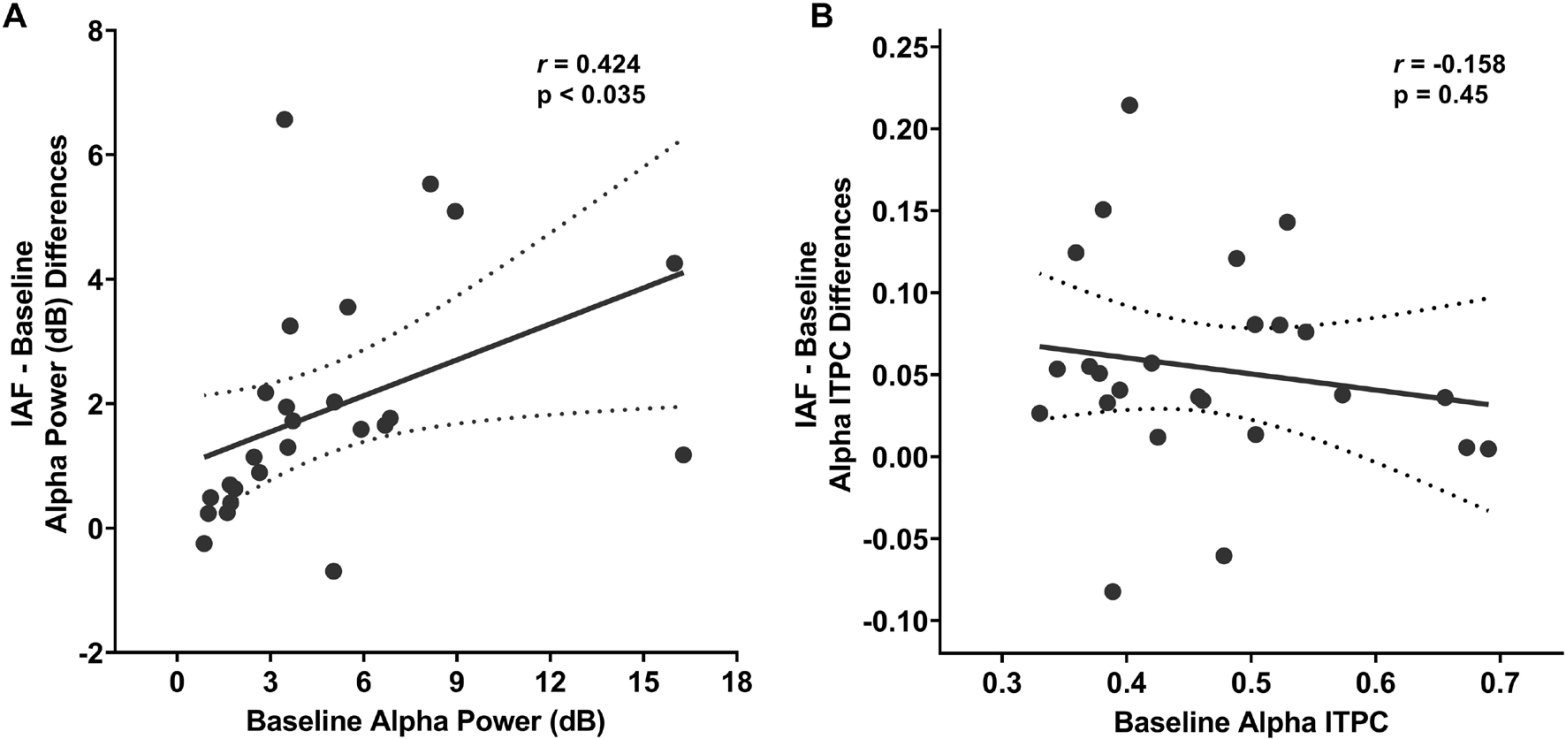
The figure highlights the relationships revealed by the Pearson correlation coefficient between baseline Alpha Power (A) and Inter‐Trial Phase Coherence (ITPC; B) and the differences observed between post‐IAF entrainment and the baseline activity. A positive association was found only for the alpha power (*r* = 0.424; *p* < 0.035).

Regarding alpha power, we found that in the posterior ROIs, higher baseline power was associated with a greater difference following IAF entrainment (*r* = 0.424, *p* = 0.035; Figure 7A), but not after IAF −2 Hz (*r* = 0.353, *p* = 0.084) or IAF +2 Hz (*r* = 0.265, *p* = 0.2). Conversely, for the anterior ROI, no significant correlations emerged (*p* > 0.65), suggesting that power modulations in anterior scalp areas may reflect the widespread entrainment‐induced modulations observed in posterior regions. On the other hand, regarding alpha ITPC, the results did not indicate significant correlations for either the posterior (*p* > 0.45; Figure 7B) or anterior ROIs (*p* > 0.62).

Overall, these correlational results suggest that increased alpha power following IAF stimulation may be predicted by higher alpha power during the baseline condition, particularly in the posterior scalp areas, with no significant associations emerging for the ITPC measure.

## 4 Discussion

The main purpose of the current study was to investigate whether prolonged alpha‐ and theta‐band visual entrainment can induce persistent and frequency‐specific modulations of resting‐state oscillatory activity following the stimulation offset, with potential differential outcomes depending on the stimulation frequency (i.e., IAF, IAF −2 Hz, IAF + 2 Hz, Theta). Additionally, we investigated whether these entrainment‐induced effects were diffuse rather than topographically focal on the scalp, inspecting neural activity across posterior, central, and anterior brain areas.

First, we found that, compared to baseline activity, prolonged alpha‐band visual entrainment selectively induced increases in power and inter‐trial phase coherence (ITPC) in the alpha, but not theta, frequency band, which persisted throughout the 1‐min post‐entrainment resting‐state period, thus suggesting that the effects of entrainment were sustained over time. Notably, entrainment of neural oscillations at participants' IAF resulted in stronger modulations of alpha spectral measures with respect to IAF −2 Hz and IAF + 2 Hz stimulation frequencies, with comparable after‐effects in both posterior scalp areas contralateral and ipsilateral to the stimulated hemifield, along with a further increase in alpha oscillatory spectral measures that extended across the central and anterior scalp regions. The pattern of results suggests that the observed post‐stimulation effects reflect a long‐lasting modulation of intrinsic oscillatory dynamics rather than sustained externally driven, stimulus‐locked activity. In particular, the individual alpha peak did not show persistent shifts toward the stimulation frequency, indicating that rhythmic stimulation interacted with the resonant properties of the endogenous oscillatory system. This interpretation aligns with recent accounts of neural entrainment emphasizing resonance‐based mechanisms rather than strict phase continuation after stimulation offset (Helfrich et al. 2019; Duecker et al. 2024).

Importantly, the observed effects were stable across the experiment and not influenced by experimental factors, as demonstrated by control analyses accounting for both session‐related variability and time‐on‐task effects, supporting robust and stable entrainment‐induced modulations. While this overall pattern of results aligns with previous studies and provides further evidence of the efficacy of visual entrainment in enhancing ongoing alpha oscillatory activity (for recent reviews, see Gallina et al. 2023; Duecker et al. 2024), our findings revealed significant aspects of sensory entrainment that can extend its potential applications by highlighting how prolonged visual rhythmic stimulation can impact the activity of the neural oscillatory system.

### 4.1 Tuning Neural Oscillations to Induce Persistent Modulations

Our findings demonstrate that the after‐effects of prolonged visual entrainment on resting‐state oscillations persist well beyond stimulation offset (i.e., up to ~1 min).

First, this pattern was evident in the alpha‐band, where sustained modulations of endogenous oscillatory activity were observed throughout the entire post‐stimulation period, as reflected by increases in both oscillatory power and phase consistency. Importantly, these effects occurred without any persistent changes in the individual alpha frequency (IAF), indicating that the observed post‐stimulation modulations of alpha oscillations do not reflect the sustained imposition of an external driving rhythm, but instead represent changes within the intrinsic resonant frequency range of the neural system. This pattern aligns with the view that entrainment after‐effects reflect resonance‐based modulation of endogenous neural oscillations rather than sustained externally driven activity following stimulation offset (Helfrich et al. 2019; Gallina et al. 2023; Duecker et al. 2024). Additionally, the absence of long‐lasting changes in IAF is consistent with previous evidence showing that entrainment can modulate spectral power and phase coherence without altering the frequency speed of the alpha rhythm, which is thought to represent a relatively stable neural feature regulated by top‐down mechanisms (Grandy et al. 2013; Wiesman and Wilson 2019; Gray and Emmanouil 2020). Taken together, these findings suggest that prolonged visual entrainment induces modulations of endogenous, ongoing oscillatory dynamics within the alpha band, rather than reflecting externally driven or stimulus‐locked activity imposed on the neural oscillatory system (Keitel et al. 2018, 2019; Gray and Emmanouil 2020). In particular, sustained increases in endogenous oscillatory power provide a robust index of long‐lasting changes in intrinsic neural activity following rhythmic stimulation (Keitel et al. 2019; Gray and Emmanouil 2020; Gallina et al. 2023; Duecker et al. 2024). Consistent with this interpretation, we observed a generalized and persistent increase in alpha‐band power following all alpha stimulation conditions (IAF, IAF −2 Hz, IAF +2 Hz), with the strongest effects emerging when stimulation was aligned with each participant's individual alpha peak, and distributed across all scalp regions examined. In addition to these power effects, we observed concurrent increases in phase consistency (ITPC) in the alpha band, selectively following IAF stimulation and predominantly over posterior scalp regions. However, within this framework, the functional significance of entrainment‐induced changes in phase consistency is not straightforward. Modulations of oscillatory phase coherence alone following visual rhythmic stimulation do not necessarily constitute direct evidence of entrainment after‐effects, given the reliance of phase consistency on external stimulation (Keitel et al. 2018, 2019; Gray and Emmanouil 2020). In the present case, given their co‐occurrence with sustained power modulations, these phase effects may reflect a persistent enhancement in the temporal coordination of endogenous neural activity within the intrinsic alpha‐band frequency range, rather than sustained phase‐locking to the previously presented stimulation (Keitel et al. 2018, 2019). Overall, the alpha‐band findings support the view that prolonged rhythmic sensory stimulation primarily interacts with the resonance properties of endogenous neural oscillators, rather than imposing a persistent externally driven rhythm (Gallina et al. 2023; Duecker et al. 2024).

A different pattern emerged in the theta band. In contrast to the alpha band, no entrainment‐induced modulation of theta power was observed following stimulation at a fixed theta frequency (4.5 Hz), suggesting that prolonged theta stimulation did not induce persistent changes in endogenous theta oscillatory activity. However, we observed a selective and sustained increase in theta‐band ITPC over central scalp regions during the post‐stimulation period. This dissociation between power and phase effects suggests that theta‐band results should be interpreted with caution, as phase synchronization alone may not be sufficient to infer sustained entrainment of intrinsic oscillatory activity (Keitel et al. 2018, 2019; Gray and Emmanouil 2020). Additionally, at the methodological level, these limited effects observed in the theta band may be explained by differences in stimulation protocols across frequencies. In the alpha band, maximal entrainment effects of endogenous oscillations were observed when stimulation was tailored to each participant's individual alpha frequency, supporting the idea that entrainment is strongest when stimulation matches the intrinsic resonance properties of neural circuits (Vossen et al. 2015; Notbohm et al. 2016). In contrast, theta stimulation was delivered at a fixed frequency. Future research employing individualized theta stimulation protocols will be important to determine whether stronger and more persistent entrainment effects of endogenous oscillatory dynamics can be observed in this frequency range.

Although prior research has consistently demonstrated short‐lasting effects (~500 ms) of brief sensory entrainment on oscillatory activity (Thut et al. 2011; Mathewson et al. 2012; Spaak et al. 2014), the neural mechanisms underlying differences between short‐ and long‐duration entrainment, as well as the persistence of after‐effects following rhythmic visual stimulation, remain poorly understood (Gallina et al. 2023; Duecker et al. 2024). In this context, a recent study by Menétrey and Pascucci (2024) reported negative after‐effects (i.e., decreases in alpha‐band power) following short periods of visual entrainment administered at a fixed frequency (10 Hz). These effects were interpreted as a compensatory adaptation or rebound mechanism, providing evidence for dedicated and flexible entrainment processes that extend beyond the simple reverberation of superimposed responses. In contrast, our prolonged visual entrainment paradigm revealed sustained increases in oscillatory activity following stimulation. This divergence suggests that the effects of entrainment may critically depend on stimulation duration. Specifically, short stimulation trains may engage rapid adaptive mechanisms leading to suppressive after‐effects, whereas prolonged stimulation may induce plasticity‐like changes in intrinsic oscillatory networks, resulting in sustained enhancements (Zaehle et al. 2010; Neuling et al. 2013; Vossen et al. 2015; Kasten et al. 2016). More broadly, these findings raise the possibility that entrainment operates across multiple timescales, with short‐term stimulation inducing adaptive recalibration of oscillatory activity and prolonged stimulation engaging longer‐lasting plastic changes in large‐scale neural dynamics. Additionally, while Menétrey and Pascucci (2024) employed a fixed stimulation frequency (10 Hz), our study used individualized stimulation frequencies (IAF), which may further contribute to the observed differences by targeting each participant's intrinsic resonance properties. Future studies combining prolonged entrainment with trial‐resolved analyses will be important to determine whether these different types of after‐effects can coexist.

Regarding the neural mechanisms underlying prolonged entrainment, it has been hypothesized that the transient modulations of neural oscillatory activity observed after short trains of rhythmic entrainment may rely on mechanisms of long‐term synaptic plasticity (LTP), which promote synchronization in the firing of the stimulated neuronal populations and enhancements in oscillatory activity (Vossen et al. 2015). Similarly, previous studies using tACS have observed long‐lasting effects in which increases in resting‐state alpha activity at the entrained frequency persist for several minutes after the offset of prolonged stimulation (Neuling et al. 2013; Vossen et al. 2015; Kasten et al. 2016). At a mechanistic level, it has been suggested that rhythmic stimulation synchronizes ongoing endogenous neural rhythms, modulates the timing of neuronal spikes, and consequently triggers spike timing‐dependent plasticity (STDP), which increases short‐ and long‐range coherence within and across brain regions (Zaehle et al. 2010; Wischnewski et al. 2023). Accordingly, it is conceivable that the persistent increases in alpha spectral measures observed in the present study could also depend on similar synaptic plasticity mechanisms. In detail, we can speculate that STDP may have strengthened oscillatory activity by aligning neuronal firing patterns with the external stimulation rhythm, inducing functional reorganization of underlying resting‐state neural oscillatory systems (Malenka and Bear 2004; Feldman 2012; Turrigiano 2012; Vossen et al. 2015). Thus, the repeated alignment of neuronal populations following rhythmic visual stimulation likely induced functional modifications, promoting long‐lasting plastic changes in the underlying resting‐state oscillatory circuits (Klimesch 2012; Vossen et al. 2015; Siebenhühner et al. 2020).

### 4.2 Widespread Spatial Effects of Prolonged Alpha‐Band Entrainment

Our findings suggest that prolonged alpha visual entrainment induces diffuse and distributed effects on alpha activity, rather than focal modulations confined to contralateral posterior areas, as previously reported (Thut et al. 2011; Mathewson et al. 2012; Spaak et al. 2014). Unlike prior studies employing short trains of visual entrainment (~0.5–5 s; Mathewson et al. 2011; De Graaf et al. 2013; Spaak et al. 2014; Kizuk and Mathewson 2017; Gray and Emmanouil 2020), our sustained rhythmic stimulation revealed increased alpha power at the entrainment offset not only in contralateral posterior areas but also in ipsilateral and centro‐anterior regions. This suggests that prolonged stimulation may engage widespread cortical networks, leading to broad alpha power increases involving plasticity mechanisms within and beyond occipito‐parietal regions, promoting reverberating circuits and diffuse cortical excitability (Vossen et al. 2015; Otero et al. 2022).

Interestingly, these widespread effects align with frameworks describing alpha oscillations as traveling waves transmitting information across brain areas (Zhang et al. 2018; Alamia and VanRullen 2019, 2024; Fakche and Dugué 2024). While alpha activity is predominantly distributed over the occipito‐parietal cortices (e.g., Williamson et al. 1997), visual stimulation can extend alpha oscillations to ipsilateral and centro‐anterior regions, propagating in multiple directions depending on brain state and task demands (Alamia and VanRullen 2024; Fakche and Dugué 2024). Accordingly, the observed alpha power increases in both ipsilateral, central and anterior scalp areas may also rely on a propagation of entrainment‐induced modulations in the contralateral posterior scalp areas. Additionally, baseline alpha power significantly predicted post‐entrainment effects selectively in posterior, but not central and anterior, scalp areas when stimulation was tuned to participants' IAF, supporting the hypothesis that the stimulation rhythm resonant with the dominant frequency of the visual system (i.e., IAF) may synchronize ongoing alpha oscillatory activity, with potential diffused effects extending to the anterior scalp areas. However, future studies are needed to explore whether prolonged alpha‐band entrainment promotes communication across broader networks by modulating alpha traveling waves observable even at the offset of the stimulation (Alamia and VanRullen 2019, 2024; Fakche and Dugué 2024).

## 5 Conclusions and Future Perspectives

Overall, our findings provide the first empirical evidence that visual entrainment can induce long‐lasting modulations of intrinsic oscillatory activity, reliably observed in the alpha, but not theta, frequency‐band when stimulation is aligned with participants' IAF. Despite the persistent post‐entrainment effects observed in our study, it remains to be clarified by which neural mechanisms the entrainment‐driven neural signal can sustain over time. As discussed in the previous section, long‐lasting entrainment effects may rely on spike‐timing‐dependent plasticity mechanisms (Vossen et al. 2015), with computational models demonstrating that maximal and sustained effects occur when the stimulation frequency aligns with the resonance frequency of the targeted neural system (Jansen et al. 1993; Otero et al. 2022). While these findings bridge models of neural oscillations with empirical evidence, future studies integrating both empirical and computational data are needed to further clarify the mechanisms underlying neural entrainment.

An intriguing direction for future research is the potential use of sensory entrainment to functionally enhance oscillatory activity. Given that alpha oscillations are closely linked to various aspects of visual perception, attention, memory, and are considered a reliable index of perceptual processing (Klimesch et al. 2007; Sadaghiani and Kleinschmidt 2016; Keller et al. 2017; Gallina et al. 2024), our findings may indicate that prolonged entrainment paradigms could selectively enhance sensory encoding and impact visual awareness through plastic reorganization of the underlying oscillatory systems. In this realm, such prolonged sensory entrainment approaches may offer valuable benefits for patients with posterior brain damage and visual field defects (Pietrelli et al. 2019; Allaman et al. 2021; Gallina, Pietrelli, et al. 2022; Gallina, Zanon, et al. 2022; Gallina et al. 2023), as well as for individuals with neurodevelopmental disorders characterized by atypical neural oscillatory activity (Ippolito et al. 2022; Marsicano et al. 2024, 2025, 2026).

## Author Contributions

G.M., J.G., L.R., C.B. – conceptualization. G.M., J.G., L.R., C.B. – data curation. G.M. – formal analysis. C.B., S.G. – funding acquisition, G.M. – investigation, G.M., J.G., L.R., C.B. – methodology. G.M. – software, visualization. C.B., L.R., S.G. – supervision. G.M., L.R., C.B. – writing – original draft preparation. G.M., J.G., S.G., L.R., C.B. – writing and review and editing.

## Funding

This work was supported by Ministero dell'Università e della Ricerca, PRIN 2017 (2017TBA4KS_003), PRIN 2022 (2022TBKCM5).

## Conflicts of Interest

The authors declare no conflicts of interest.

## Supporting information


**Figure S1:** Temporal dynamics of entrainment effects on oscillatory power and inter‐trial phase coherence (ITPC). Second‐by‐second time course of oscillatory activity across the 60‐s post‐stimulation resting‐state period for representative regions of interest (ROIs) in which strong entrainment effects were observed. (A) Temporal evolution of oscillatory power in the alpha (8–13 Hz) (left panel; posterior ROI) and theta (3–6 Hz) bands (right panel; posterior ROI). (B) Temporal evolution of inter‐trial phase coherence (ITPC) in the alpha band (left panel; posterior ROI) and theta band (right panel; anterior ROI). Lines represent the mean values across participants for each stimulation condition (Baseline, IAF, IAF −2 Hz, IAF +2 Hz, Theta), and shaded areas indicate the standard error of the mean (SEM). The temporal profiles illustrate that the modulation of oscillatory power and ITPC following rhythmic stimulation persists across the entire post‐stimulation resting‐state interval rather than decaying rapidly within the first seconds after stimulation offset, supporting the interpretation of sustained entrainment‐related effects rather than short‐lived transient responses.
**Figure S2:** Stability of oscillatory activity across the nine post‐stimulation resting‐state blocks. (A) Mean oscillatory power in the alpha (8–13 Hz) and theta (3–6 Hz) bands across the nine resting‐state blocks following each stimulation condition (Baseline, IAF, IAF −2 Hz, IAF +2 Hz, Theta), shown separately for posterior, central, and anterior regions of interest (ROIs). (B) Mean inter‐trial phase coherence (ITPC) in the alpha (8–13 Hz) and theta (3–6 Hz) bands across the nine resting‐state blocks for the same stimulation conditions and ROIs. Lines represent the mean values across participants and shaded areas indicate the standard error of the mean (SEM). The figure illustrates that oscillatory power and ITPC remained largely stable across resting‐state blocks for all stimulation conditions, supporting the absence of systematic time‐on‐task effects across the experimental session.
**Table S1:**. Results of the repeated‐measures ANOVAs conducted after excluding the initial portion of the resting‐state post‐stimulation period (first 3000 ms) to control for potential transient responses immediately following rhythmic stimulation. Analyses were performed separately for oscillatory power and inter‐trial phase coherence (ITPC) within the alpha (8–13 Hz) and theta (3–6 Hz) frequency bands across the three regions of interest (posterior, central, anterior). The ANOVAs included Condition (Baseline, IAF, IAF −2 Hz, IAF +2 Hz, Theta), Temporal Segment, Hemisphere, and Stimulated Hemifield as within‐subject factors. The results replicate the pattern observed in the main analyses, with significant Condition effects observed in the same ROIs and measures, indicating that the entrainment‐related modulations of oscillatory power and phase coherence remain present after excluding the initial post‐stimulation interval. Significant effects are marked with an asterisk (*p < 0.05).
**Table S2:** Results of the repeated‐measures ANOVAs assessing the stability of entrainment effects across the nine post‐stimulation resting‐state blocks. Analyses were conducted separately for power and inter‐trial phase coherence (ITPC) measures within the alpha (8–13 Hz) and theta (3–6 Hz) frequency bands and across the three regions of interest (ROIs: posterior, central, anterior). For each analysis, the effects of Condition (Baseline, IAF, IAF −2 Hz, IAF +2 Hz, Theta), Resting‐State Block (nine levels), and their interaction (Condition × Resting‐State Block) are reported. Overall, the analyses replicated the main effects of Condition previously described, while no significant Resting‐State Block effects or Condition × Resting‐State Block interactions were observed, indicating that the magnitude of the entrainment effects remained stable across the Resting‐state Blocks. The only exception was a significant main effect of Resting‐State Block for theta power in the anterior ROI. Significant effects are marked with an asterisk (*p < 0.05).

## Data Availability

The data that support the findings of this study are available from the corresponding author upon reasonable request.
