# Supplementary material for: Prolonged Visual Entrainment Induces Long‐Lasting Alpha‐Band Modulations
Source: Psychophysiology. 2026 May 17;63:e70321. doi: 10.1111/psyp.70321 (PMC13181227; doi:10.1111/psyp.70321)
Supplement: Supplementary file 1 — Figure S1: Temporal dynamics of entrainment effects on oscillatory power and inter‐trial phase coherence (ITPC). Second‐by‐second time course of oscillatory activity across the 60‐s post‐stimulation resting‐state period for representative regions of interest (ROIs) in which strong entrainment effects were observed. (A) Temporal evolution of oscillatory power in the alpha (8–13 Hz) (left panel; posterior ROI) and theta (3–6 Hz) bands (right panel; posterior ROI). (B) Temporal evolution of inter‐trial phase coherence (ITPC) in the alpha band (left panel; posterior ROI) and theta band (right panel; anterior ROI). Lines represent the mean values across participants for each stimulation condition (Baseline, IAF, IAF −2 Hz, IAF +2 Hz, Theta), and shaded areas indicate the standard error of the mean (SEM). The temporal profiles illustrate that the modulation of oscillatory power and ITPC following rhythmic stimulation persists across the entire post‐stimulation resting‐state interval rather than decaying rapidly within the first seconds after stimulation offset, supporting the interpretation of sustained entrainment‐related effects rather than short‐lived transient responses. Figure S2: Stability of oscillatory activity across the nine post‐stimulation resting‐state blocks. (A) Mean oscillatory power in the alpha (8–13 Hz) and theta (3–6 Hz) bands across the nine resting‐state blocks following each stimulation condition (Baseline, IAF, IAF −2 Hz, IAF +2 Hz, Theta), shown separately for posterior, central, and anterior regions of interest (ROIs). (B) Mean inter‐trial phase coherence (ITPC) in the alpha (8–13 Hz) and theta (3–6 Hz) bands across the nine resting‐state blocks for the same stimulation conditions and ROIs. Lines represent the mean values across participants and shaded areas indicate the standard error of the mean (SEM). The figure illustrates that oscillatory power and ITPC remained largely stable across resting‐state blocks for all stimulation conditions, suppo [file PSYP-63-e70321-s001.docx]

**Supplementary Materials**

**Supplementary Results**

**Consistency and Stability of Entrainment Effects**

An additional control analysis was performed to rule out potential confounds related to transient post-stimulation responses. Specifically, all analyses were repeated after excluding the first 3000 ms of the post-stimulation period. The results replicated the main findings (see main text Results section), with significant effects of Condition on oscillatory power and ITPC remaining unchanged across ROIs and frequency bands (see Table S1), indicating that the observed modulations were not driven by transient activity. Here, we report in detail all effects and post-hoc comparisons from these control ANOVAs.

***Alpha-band (7-13 Hz) power***

For the posterior ROIs, the ANOVA performed on alpha power revealed significant main effects of Temporal Segment (F(2, 48) = 12.20, p < .001), Hemisphere (F(1, 24) = 4.99, p = 0.035), and Condition (F(4,96) = 15.04, p < 0.001). Post-hoc comparisons for Temporal Segment indicated that alpha power was higher in the first segment compared to the second (p = 0.032) and third (p < 0.001) segments, with a further decrease in alpha power observed from the second to the third segment (p = 0.044). Post-hoc comparisons for Hemisphere showed that alpha power was higher in the right hemisphere with respect to the left (p = 0.043). Regarding Condition main factor, post-hoc comparisons revealed that alpha power was significantly higher following IAF (p < 0.001), IAF -2 Hz (p < 0.001), and IAF +2 Hz ( p = 0.001), as compared to the baseline condition. However, alpha power following Theta stimulation did not significantly differ from baseline (p = 0.35), confirming that only IAF-range entrainment effectively increased alpha power. In addition, Theta condition showed a lower alpha power with respect to IAF (p < 0.001) and IAF -2 Hz (p = 0.16) conditions. No other significant main effects and interactions were found (p-values > 0.15), supporting that the effects induced by the alpha-band entrainment on alpha power were independent of the temporal segment, hemisphere, and stimulated hemifield. For the central ROIs, the ANOVA performed on the alpha power revealed significant main effects of Temporal Segment (F(2, 48) = 8.88, p < 0.001) and Condition (F(4, 96) = 3.26, p = .014). Post-hoc comparisons for the Temporal Segment indicated that alpha power was higher in the first segment compared to the third segment (p < 0.001). Regarding Condition main factor, post-hoc comparisons revealed that alpha power was significantly increased following IAF as compared to baseline (p = 0.011) and theta (p = 0.037) conditions. For the anterior ROI, the ANOVA performed on the alpha power revealed significant main effects of Temporal Segment (F(2, 48) = 4.14, p = 0.021) and Condition (F(4, 96) = 4.93, p = .001). Post-hoc comparisons for the Temporal Segment indicated that alpha power was higher in the first segment compared to the third segment (p = 0.006). Regarding Condition main factor, post-hoc comparisons revealed that alpha power was significantly increased following IAF (p = 0.004) and IAF+2 Hz (p = 0.018) with respect to the baseline condition, with a further significant power difference between IAF and Theta stimulation condition (p = 0.027).

***Theta-band (3-6 Hz) power***

In contrast to the findings for alpha power, the control ANOVA conducted on theta power over the posterior ROIs revealed a significant main effect of Hemisphere (F(1, 24) = 5.58, p = 0.026), with higher theta power observed in the right hemisphere compared to the left hemisphere (p = 0.0267). No other significant main effects or interactions were found (p-values > 0.061). For the central ROIs, the ANOVA revealed a significant main effect of Hemisphere (F(1, 24) = 5.63, p = 0.025), with higher theta power observed in the right hemisphere compared to the left hemisphere (p = 0.026). In the anterior ROI, no significant main effects or interactions were observed (p-values > 0.119).

***Alpha-band (7-13 Hz) ITPC***

For the posterior ROIs, the control analyses ANOVA performed on alpha ITPC revealed significant main effects of Temporal Segment (F(2, 48) = 5.18, p = 0.009) and Condition (F(4,96) = 3.43, p = 0.011). Post-hoc comparisons for the Temporal Segment indicated that alpha ITPC was higher in the first segment compared to the third segment (p = 0.007). Post-hoc comparisons of the Condition factor revealed significantly higher ITPC following IAF stimulation compared to the baseline condition (p = 0.005). No other significant main effects and interactions were found (p-values > 0.086). For the central ROI, the ANOVA performed on alpha ITPC revealed significant main effects of Temporal Segment (F(2, 48) = 12.23, p < 0.001). Post-hoc comparisons for the Temporal Segment indicated that alpha ITPC was lower in the first segment compared to the second (p = 0.0011) and third segment (p < 0.001). On the other hand, in the anterior ROI, the ANOVA conducted on alpha ITPC revealed no significant main effects or interactions (p-values > 0.0592).

***Theta-band (3-6 Hz) ITPC***

The ANOVA performed on theta ITPC showed a significant main effect of Stimulated Hemifield (F(1, 24) = 4.86, p = 0.037) and Condition (F(4, 96) = 2.91, p = 0.025). Post-hoc comparisons of Stimulated Hemifield revealed a higher theta ITPC following stimulation delivered in the right hemifield as compared to the left one (p = 0.037). Regarding the Condition main effect, such analysis did not show significant post-hoc comparison among stimulation conditions (p-values > 0.076). No other significant main effects and interactions were found (p-values > 0.0596). For the central ROI, the ANOVA performed on the theta ITPC revealed a significant main effect of Hemisphere (F(1, 24) = 11.37, p = 0.002), Temporal Segment (F(2, 48) = 7.27, p < 0.001) and Condition (F(4, 96) = 3.31, p = 0.013). Post-hoc comparisons of Hemisphere revealed a higher theta ITPC in the left hemisphere as compared to the right one (p = 0.002). Post-hoc comparisons of Temporal Segments revealed a higher theta ITPC in the first Temporal Segment as compared to the second (p = 0.003) and third one (p = 0.008). Regarding the Condition main effect, post-hoc comparisons revealed a theta ITPC increase following theta entrainment with respect to the ITPC observed during the baseline condition (p = 0.0059). For the anterior ROI, the ANOVA performed on the theta ITPC did not reveal a significant main effect On the other hand, in the anterior ROI, the ANOVA conducted on theta ITPC revealed no significant main effects or interactions (p-values > 0.0535).

**Supplementary Figure 1**


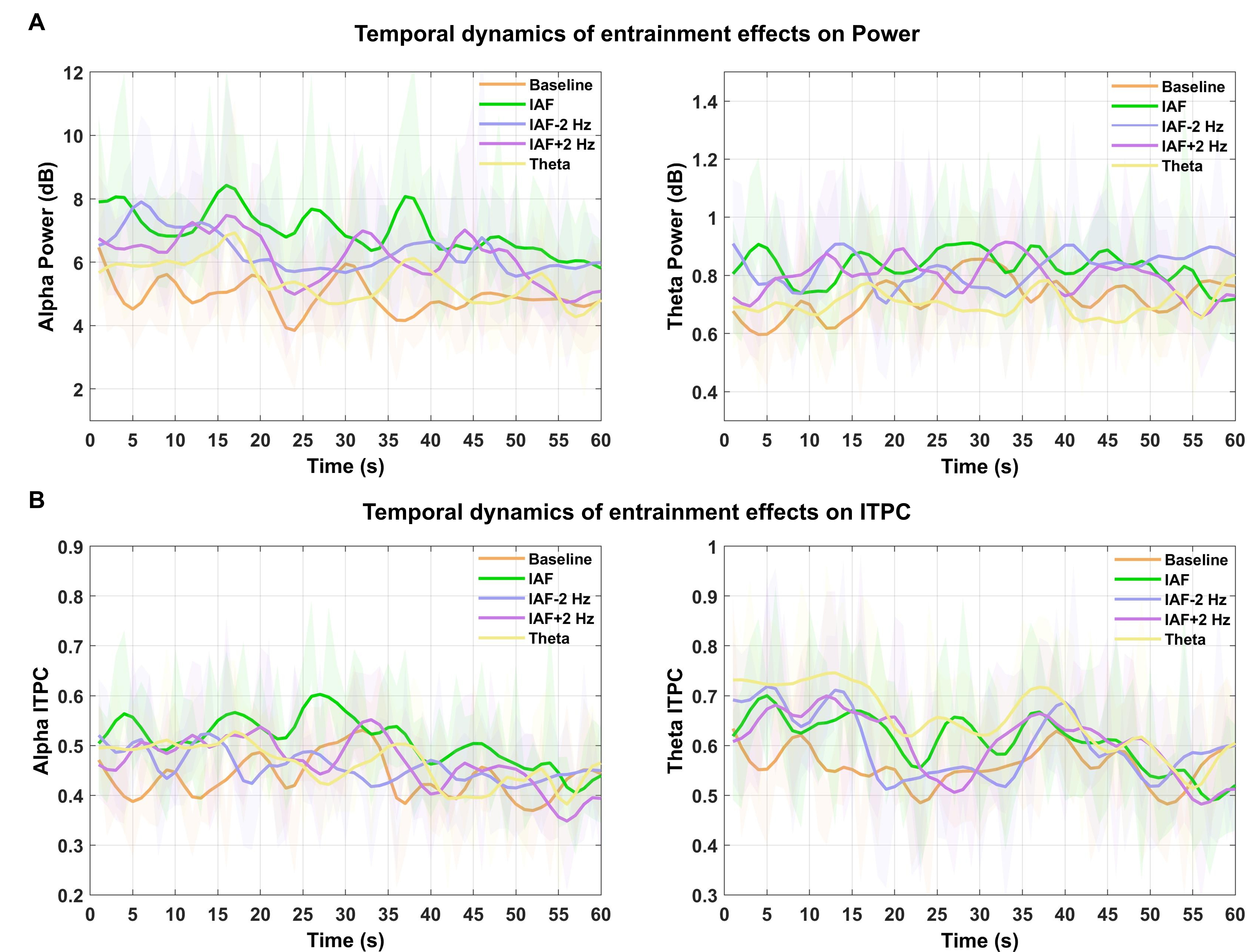


**Figure S1.** Temporal dynamics of entrainment effects on oscillatory power and inter-trial phase coherence (ITPC). Second-by-second time course of oscillatory activity across the 60-s post-stimulation resting-state period for representative regions of interest (ROIs) in which strong entrainment effects were observed. (A) Temporal evolution of oscillatory power in the alpha (8–13 Hz) (left panel; posterior ROI) and theta (3–6 Hz) bands (right panel; posterior ROI). (B) Temporal evolution of inter-trial phase coherence (ITPC) in the alpha band (left panel; posterior ROI) and theta band (right panel; anterior ROI). Lines represent the mean values across participants for each stimulation condition (Baseline, IAF, IAF−2 Hz, IAF+2 Hz, Theta), and shaded areas indicate the standard error of the mean (SEM). The temporal profiles illustrate that the modulation of oscillatory power and ITPC following rhythmic stimulation persists across the entire post-stimulation resting-state interval rather than decaying rapidly within the first seconds after stimulation offset, supporting the interpretation of sustained entrainment-related effects rather than short-lived transient responses.





**Figure S2.** Stability of oscillatory activity across the nine post-stimulation resting-state blocks. (A) Mean oscillatory power in the alpha (8–13 Hz) and theta (3–6 Hz) bands across the nine resting-state blocks following each stimulation condition (Baseline, IAF, IAF−2 Hz, IAF+2 Hz, Theta), shown separately for posterior, central, and anterior regions of interest (ROIs). (B) Mean inter-trial phase coherence (ITPC) in the alpha (8–13 Hz) and theta (3–6 Hz) bands across the nine resting-state blocks for the same stimulation conditions and ROIs. Lines represent the mean values across participants and shaded areas indicate the standard error of the mean (SEM). The figure illustrates that oscillatory power and ITPC remained largely stable across resting-state blocks for all stimulation conditions, supporting the absence of systematic time-on-task effects across the experimental session.

**Supplementary Table 1**

|  | | | | **Frequency-Band** | **Measure** | **ROIs** | **rm-ANOVA Effect** | **F** | **p-value** |
| --- | --- | --- | --- | --- | --- | --- | --- | --- | --- |
|  | | | | Alpha (8-13 Hz) | Power | Posterior | Condition | 15.04 | 0.001* |
|  | | | | Alpha (8-13 Hz) | Power | Posterior | Temporal Segment | 12.20 | 0.001* |
|  | | | | Alpha (8-13 Hz) | Power | Posterior | Hemisphere | 4.99 | 0.035* |
|  | | | | Alpha (8-13 Hz) | Power | Posterior | Stimulated Hemifield | 0.19 | 0.658 |
|  | | | | Alpha (8-13 Hz) | Power | Central | Condition | 3.26 | 0.014* |
|  | | | | Alpha (8-13 Hz) | Power | Central | Temporal Segment | 8.88 | 0.001* |
|  | | | | Alpha (8-13 Hz) | Power | Central | Hemisphere | 5.21 | 0.031* |
|  | | | | Alpha (8-13 Hz) | Power | Central | Stimulated Hemifield | 0.16 | 0.691 |
|  | | | | Alpha (8-13 Hz) | Power | Anterior | Condition | 4.93 | 0.001* |
|  | | | | Alpha (8-13 Hz) | Power | Anterior | Temporal Segment | 4.14 | 0.021* |
|  | | | | Alpha (8-13 Hz) | Power | Anterior | Stimulated Hemifield | 0.39 | 0.535 |
|  | | | | Theta (3-6 Hz) | Power | Posterior | Condition | 2.35 | 0.059 |
|  | | | | Theta (3-6 Hz) | Power | Posterior | Temporal Segment | 0.36 | 0.696 |
|  | | | | Theta (3-6 Hz) | Power | Posterior | Hemisphere | 5.58 | 0.026* |
|  | | | | Theta (3-6 Hz) | Power | Posterior | Stimulated Hemifield | 0.002 | 0.958 |
|  | | | | Theta (3-6 Hz) | Power | Central | Condition | 1.78 | 0.13 |
|  | | | | Theta (3-6 Hz) | Power | Central | Temporal Segment | 1.25 | 0.29 |
|  | | | | Theta (3-6 Hz) | Power | Central | Hemisphere | 5.63 | 0.025* |
|  | | | | Theta (3-6 Hz) | Power | Central | Stimulated Hemifield | 0.22 | 0.635 |
|  | | | Theta (3-6 Hz) | | Power | Anterior | Condition | 0.04 | 0.99 |
|  | | | Theta (3-6 Hz) | | Power | Anterior | Temporal Segment | 0.37 | 0.68 |
|  | | | Theta (3-6 Hz) | | Power | Anterior | Stimulated Hemifield | 0.40 | 0.53 |
|  | | Alpha (8-13 Hz) | | | ITPC | Posterior | Condition | 3.43 | 0.011* |
|  | | Alpha (8-13 Hz) | | | ITPC | Posterior | Temporal Segment | 5.18 | 0.009* |
|  | | Alpha (8-13 Hz) | | | ITPC | Posterior | Hemisphere | 0.80 | 0.037 |
|  | | Alpha (8-13 Hz) | | | ITPC | Posterior | Stimulated Hemifield | 0.69 | 0.41 |
|  | | | Alpha (8-13 Hz) | | ITPC | Central | Condition | 0.61 | 0.65 |
|  | | | | Alpha (8-13 Hz) | ITPC | Central | Temporal Segment | 12.23 | 0.001* |
|  | | | | Alpha (8-13 Hz) | ITPC | Central | Hemisphere | 0.23 | 0.629 |
|  | | | | Alpha (8-13 Hz) | ITPC | Central | Stimulated Hemifield | 0.003 | 0.987 |
|  | | | | Alpha (8-13 Hz) | ITPC | Anterior | Condition | 1.06 | 0.37 |
|  | | | | Alpha (8-13 Hz) | ITPC | Anterior | Temporal Segment | 2.18 | 0.12 |
|  | | | | Alpha (8-13 Hz) | ITPC | Anterior | Stimulated Hemifield | 0.91 | 0.34 |
|  | Theta (3-6 Hz) | | | | ITPC | Posterior | Condition | 2.91 | 0.025* |
|  | Theta (3-6 Hz) | | | | ITPC | Posterior | Temporal Segment | 2.99 | 0.059 |
|  | Theta (3-6 Hz) | | | | ITPC | Posterior | Hemisphere | 3.29 | 0.081 |
|  | Theta (3-6 Hz) | | | | ITPC | Posterior | Stimulated Hemifield | 4.86 | 0.037* |
|  | | | | Theta (3-6 Hz) | ITPC | Central | Condition | 3.31 | 0.013* |
|  | | | | Theta (3-6 Hz) | ITPC | Central | Temporal Segment | 7.27 | 0.001* |
|  | | | | Theta (3-6 Hz) | ITPC | Central | Hemisphere | 11.37 | 0.002* |
|  | | | | Theta (3-6 Hz) | ITPC | Central | Stimulated Hemifield | 1.57 | 0.221 |
| Theta (3-6 Hz) | | | | | ITPC | Anterior | Condition | 1.40 | 0.238 |
| Theta (3-6 Hz) | | | | | ITPC | Anterior | Temporal Segment | 3.11 | 0.053 |
| Theta (3-6 Hz) | | | | | ITPC | Anterior | Stimulated Hemifield | 1.008 | 0.325 |

**Table S1**. Results of the repeated-measures ANOVAs conducted after excluding the initial portion of the resting-state post-stimulation period (first 3000 ms) to control for potential transient responses immediately following rhythmic stimulation. Analyses were performed separately for oscillatory power and inter-trial phase coherence (ITPC) within the alpha (8–13 Hz) and theta (3–6 Hz) frequency bands across the three regions of interest (posterior, central, anterior). The ANOVAs included Condition (Baseline, IAF, IAF−2 Hz, IAF+2 Hz, Theta), Temporal Segment, Hemisphere, and Stimulated Hemifield as within-subject factors. The results replicate the pattern observed in the main analyses, with significant Condition effects observed in the same ROIs and measures, indicating that the entrainment-related modulations of oscillatory power and phase coherence remain present after excluding the initial post-stimulation interval. Significant effects are marked with an asterisk (*p < .05).

**Supplementary Table 2**

| **Frequency-Band** | **Measure** | **ROIs** | **rm-ANOVA Effect** | **F** | **p-value** |
| --- | --- | --- | --- | --- | --- |
|  |  |  |  |  |  |
| Alpha (8-13 Hz) | Power | Posterior | Condition | 15.84 | 0.001* |
| Alpha (8-13 Hz) | Power | Posterior | Resting Block | 1.44 | 0.18 |
| Alpha (8-13 Hz) | Power | Posterior | Condition – Resting Block | 1.01 | 0.44 |
| Alpha (8-13 Hz) | Power | Central | Condition | 3.30 | 0.013* |
| Alpha (8-13 Hz) | Power | Central | Resting Block | 1.06 | 0.38 |
| Alpha (8-13 Hz) | Power | Central | Condition – Resting Block | 0.95 | 0.53 |
| Alpha (8-13 Hz) | Power | Anterior | Condition | 5.20 | 0.001* |
| Alpha (8-13 Hz) | Power | Anterior | Resting Block | 0.66 | 0.73 |
| Alpha (8-13 Hz) | Power | Anterior | Condition – Resting Block | 1.39 | 0.073 |
| Theta (3-6 Hz) | Power | Posterior | Condition | 1.92 | 0.11 |
| Theta (3-6 Hz) | Power | Posterior | Resting Block | 1.47 | 0.16 |
| Theta (3-6 Hz) | Power | Posterior | Condition – Resting Block | 0.89 | 0.63 |
| Theta (3-6 Hz) | Power | Central | Condition | 1.85 | 0.012 |
| Theta (3-6 Hz) | Power | Central | Resting Block | 1.34 | 0.21 |
| Theta (3-6 Hz) | Power | Central | Condition – Resting Block | 0.90 | 0.61 |
| Theta (3-6 Hz) | Power | Anterior | Condition | 1.39 | 0.24 |
| Theta (3-6 Hz) | Power | Anterior | Resting Block | 3.41 | 0.001* |
| Theta (3-6 Hz) | Power | Anterior | Condition – Resting Block | 1.21 | 0.19 |
| Alpha (8-13 Hz) | ITPC | Posterior | Condition | 3.51 | 0.01* |
| Alpha (8-13 Hz) | ITPC | Posterior | Resting Block | 1.36 | 0.21 |
| Alpha (8-13 Hz) | ITPC | Posterior | Condition – Resting Block | 1.12 | 0.29 |
| Alpha (8-13 Hz) | ITPC | Central | Condition | 0.572 | 0.68 |
| Alpha (8-13 Hz) | ITPC | Central | Resting Block | 0.576 | 0.79 |
| Alpha (8-13 Hz) | ITPC | Central | Condition – Resting Block | 1.16 | 0.24 |
| Alpha (8-13 Hz) | ITPC | Anterior | Condition | 1.09 | 0.36 |
| Alpha (8-13 Hz) | ITPC | Anterior | Resting Block | 1.24 | 0.27 |
| Alpha (8-13 Hz) | ITPC | Anterior | Condition – Resting Block | 0.94 | 0.54 |
| Theta (3-6 Hz) | ITPC | Posterior | Condition | 2.72 | 0.033* |
| Theta (3-6 Hz) | ITPC | Posterior | Resting Block | 1.09 | 0.36 |
| Theta (3-6 Hz) | ITPC | Posterior | Condition – Resting Block | 0.77 | 0.81 |
| Theta (3-6 Hz) | ITPC | Central | Condition | 3.41 | 0.011* |
| Theta (3-6 Hz) | ITPC | Central | Resting Block | 1.55 | 0.14 |
| Theta (3-6 Hz) | ITPC | Central | Condition – Resting Block | 0.87 | 0.27 |
| Theta (3-6 Hz) | ITPC | Anterior | Condition | 1.33 | 0.26 |
| Theta (3-6 Hz) | ITPC | Anterior | Resting Block | 0.90 | 0.51 |
| Theta (3-6 Hz) | ITPC | Anterior | Condition – Resting Block | 1.18 | 0.22 |

**Table S2.** Results of the repeated-measures ANOVAs assessing the stability of entrainment effects across the nine post-stimulation resting-state blocks. Analyses were conducted separately for power and inter-trial phase coherence (ITPC) measures within the alpha (8–13 Hz) and theta (3–6 Hz) frequency bands and across the three regions of interest (ROIs: posterior, central, anterior). For each analysis, the effects of Condition (Baseline, IAF, IAF−2 Hz, IAF+2 Hz, Theta), Resting-State Block (nine levels), and their interaction (Condition × Resting-State Block) are reported. Overall, the analyses replicated the main effects of Condition previously described, while no significant Resting-State Block effects or Condition × Resting-State Block interactions were observed, indicating that the magnitude of the entrainment effects remained stable across the Resting-state Blocks. The only exception was a significant main effect of Resting-State Block for theta power in the anterior ROI. Significant effects are marked with an asterisk (*p < .05).
